# Chronic Inflammation Pathway NF-κB Cooperates with Epigenetic Reprogramming to Drive the Malignant Progression of Glioblastoma

**DOI:** 10.7150/ijbs.73749

**Published:** 2022-09-21

**Authors:** Kefeng Lin, Wenli Gao, Ning Chen, Shuyao Yang, Han Wang, Ran Wang, Fang Xie, Jiaqi Meng, Eric W.-F. Lam, Suyi Li, Wei Cheng, Puxiang Chen, Hongjin Wu, Jinsong Yan, Di Jin, Bilian Jin

**Affiliations:** 1 Institute of Cancer Stem Cell; Liaoning Key Laboratory of Nucleic Acid Biology, Dalian Medical University, Dalian 116044, Liaoning, China.; 2 Department of Hematology; Liaoning Key Laboratory of Hematopoietic Stem Cell Transplantation and Translational Medicine; Liaoning Medical Center for Hematopoietic Stem Cell Transplantation; Dalian Key Laboratory of Hematology; Diamond Bay Institute of Hematology, Second Hospital of Dalian Medical University, Dalian 116044, China.; 3 Boao International Hospital, Shanghai University of Traditional Chinese Medicine, Qionghai 571734, Hainan, China.; 4 Department of Obstetrics and Gynecology, The Second Xiangya Hospital, Central South University, Changsha 410011, Hunan, China.

## Abstract

Without an effective strategy for targeted therapy, glioblastoma is still incurable with a median survival of only 15 months. Both chronic inflammation and epigenetic reprogramming are hallmarks of cancer. However, the mechanisms and consequences of their cooperation in glioblastoma remain unknown. Here, we discover that chronic inflammation governs H3K27me3 reprogramming in glioblastoma through the canonical NF-κB pathway to target EZH2. Being a crucial mediator of chronic inflammation, the canonical NF-κB signalling specifically directs the expression and redistribution of H3K27me3 but not H3K4me3, H3K9me3 and H3K36me3. Using RNA-seq screening to focus on genes encoding methyltransferases and demethylases of histone, we identify EZH2 as a key methyltransferase to control inflammation-triggered epigenetic reprogramming in gliomagenesis. Mechanistically, NF-κB selectively drives the expression of EZH2 by activating its transcription, consequently resulting in a global change in H3K27me3 expression and distribution. Furthermore, we find that co-activation of NF-κB and EZH2 confers the poorest clinical outcome, and that the risk for glioblastoma can be accurately molecularly stratified by NF-κB and EZH2. It is notable that NF-κB can potentially cooperate with EZH2 in more than one way, and most importantly, we demonstrate a Synergistic effect of cancer cells induced by combinatory inhibition of NF-κB and EZH2, which both are frequently over-activated in glioblastoma. In summary, we uncover a functional cooperation between chronic inflammation and epigenetic reprogramming in glioblastoma, combined targeting of which by inhibitors guaranteed in safety and availability furnishes a potent strategy for effective treatment of this fatal disease.

## Introduction

Glioblastoma, the most aggressive tumour in the central nervous system, is still incurable with an intermediate survival of only 15 months 1, 2. In past decades, great efforts have been made to search for appropriate targets for therapy. So far, however, no effective strategy of targeted therapy has been developed in the clinic.

Previous researches have suggested that chronic inflammation is a cardinal feature of glioblastoma biology 3, 4. The blood-brain barrier is destroyed by glioblastoma, causing chronic neuroinflammation 5, 6. The inflammatory microenvironment is an essential component of the vast majority of tumours to drive their malignant progression 7, 8. In consequence, the mechanisms connecting inflammation to tumorigenesis have been a primary focus of cancer research 7, 9. NF-κB is a key transcription factor linking chronic inflammation and cancer 10, 11. Solid and direct genetic evidence showed that the NF-κB signalling is an essential mediator of tumour promotion 12, 13. Similar to other carcinomas, glioblastoma displays high constitutive NFκB activity, and various central tumorigenic signaling pathways of glioblastoma converge on NFκB 14, 15.

Epigenetic modifications are central to cancer 16, 17. Histone lysine methylation is one of the best-characterized histone modifications 18, 19. The function of lysine methylation depends on its site of residue and the degree of methylation (mono, di or trimethylation) in the tail structure 20. The most widely researched histone methylation loci comprise of H3K4, H3K9, H3K27, and H3K36, etc. 18, 21. H3K27me3 is recognized as a vital epigenetic modification during differentiation and proliferation 22, and dysregulation of which was constantly observed in many kinds of tumours, including glioblastoma 23, 24.

Both chronic inflammation and epigenetic reprogramming are hallmarks of cancer 25. However, whether there exists a functional cooperation between them in glioblastoma remains unclear. Here, we discover that the NF-κB activation dictates the methylation of H3K27, and we identify EZH2 as an essential methyltransferase to control the reprogramming of histone modification. Significantly, co-activation of NF-κB and EZH2 confers the poorest clinic outcome and glioblastoma can be molecularly stratified by NF-κB and EZH2. Most importantly, cancer cells are induced to a Synergistic effect by combinatory inhibition of NF-κB and EZH2. Both NF-κB and EZH2 have long been regarded as crucial targets for cancer treatment. However, targeting eath of them alone has never been successful in clinic except that several inhibitors are guaranteed in safety and availability. Combined targeting of them by pharmaceutical interventions uncovers a novel potent strategy for glioblastoma patients.

## Results

### Canonical NF-κB signalling specifically activates global methylation of H3K27 in glioblastoma

**Figure 1:**
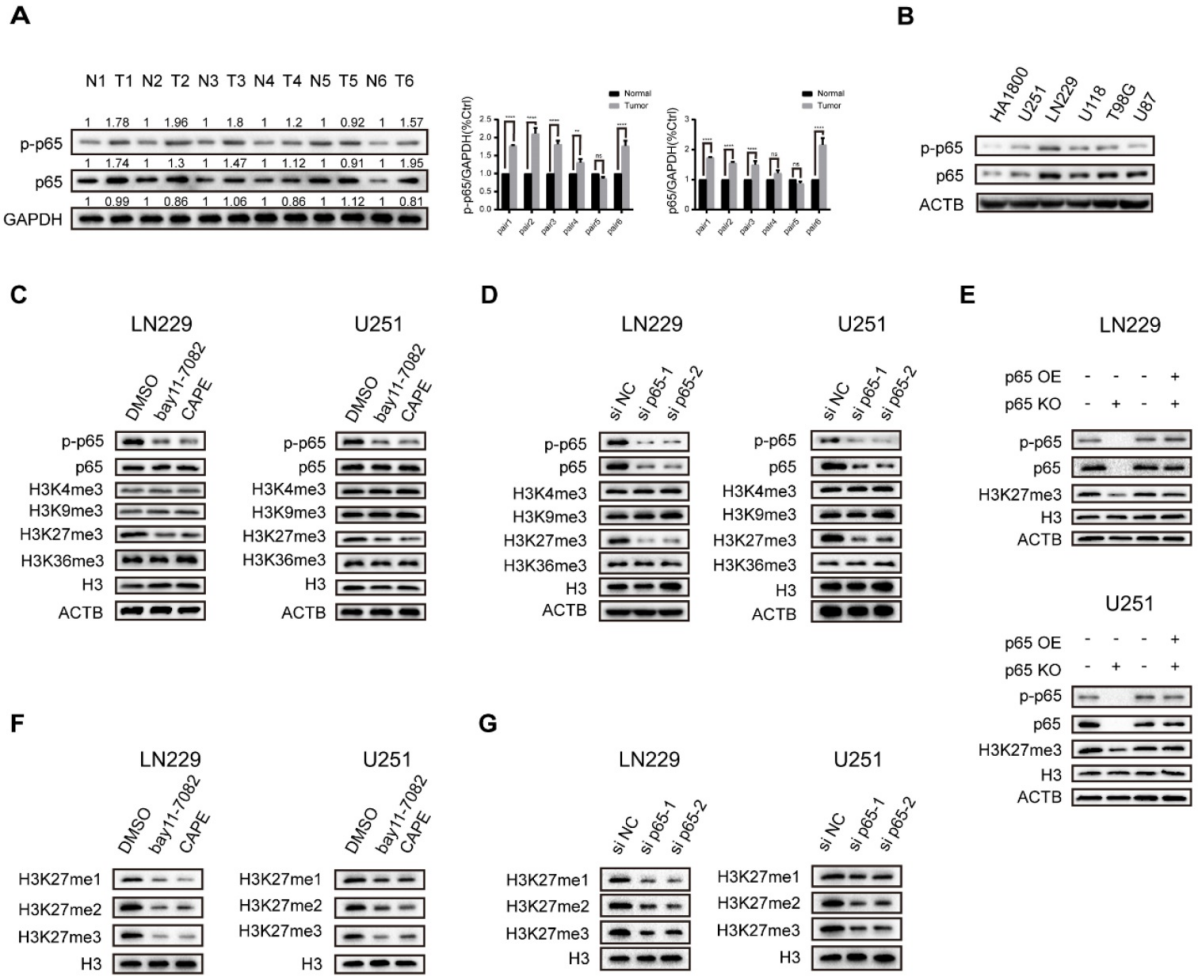
**Canonical NF-κB signalling specifically activates global methylation of H3K27 in glioblastoma. a** NF-κB activation status in glioblastoma samples. Results showed by western blotting. **b** Protein expression of p65 and p-p65 in five general glioblastoma cell lines. Results showed by western blotting.** c** The LN229 and U251 cells were cultured in 5 µM bay11-7082 or 3 µM CAPE. Levels of histone lysine methylation were detected by western blotting.** d** LN229 and U251 cells were transfected with si NC oligos or si p65 oligos. Levels of histone lysine methylation were detected by western blotting.** e** Rescue experiment performed in LN229 and U251 cells. Results showed by western blotting. **f, g** The expression levels of H3K27me1/2/3 upon knockdown p65 or NF-κB inhibitors in LN229 and U251 cells. Results showed by western blotting. Values are expressed as the means±SD from three experiments

Recent research discovered that NF-κB, the key transcription factor linking chronic inflammation with cancer 26, displays constitutive activity in primary glioblastoma where phosphorylated and total p65 expression is significantly upregulated 27. To confirm that, we examined the status of NF-κB activation in 6 pairs of glioblastoma tumour and normal samples and five general glioma cell lines, and the results suggested that their expression is frequently upregulated in glioblastoma (Fig. 1a, b). The LN229 cells were found to have relatively high p65 expression, while U251 was found to have comparatively low p65 expression (Fig. 1b). Thus, we chose LN229 and U251 for further studies.

Histone lysine methylation plays a critical role in epigenetic modification and transcription 20. The most extensively studied histone methylation sites include H3K4, H3K9, H3K27, H3K36, etc. 18. Previous research reported that NF-κB directly mediates epigenetic deregulation in B-cells and lymphomas 28. Therefore, we sought to explore the impacts of NFκB on trimethylation of H3 residues. To establish a system to monitor the impact of NF-κB activation, we examined NF-κB-activated luciferase values in the treatment of both NF-κB activators and inhibitors. The results showed that the NF-κB activators LPS and TNF-α promoted and the inhibitors bay11-7082 and CAPE repressed NF-κB-driven transcriptional activity dose-dependently in both LN229 and U251 cell lines (Fig. S1a, b), validating the ability of our luciferase reporter gene assay to monitor NF-κB activity in glioma cell lines. Then we studied their effects on H3K4-, H3K9-, H3K27 and H3K36-trimethylation using Western blot analysis. The NF-κB activators and inhibitors caused a change in the tri-methylation levels of H3K27 in both cell lines. In contrast, the tri-methylation levels of H3K4, H3K9 and H3K36 were largely unaffected (Fig. 1c and Fig. S1c). Next, we utilized siRNA and overexpression vector to deplete and increase p65 expression respectively in LN229 and U251 cells. The results are consistent with the chemical activators and inhibitors (Fig. 1d and Fig. S1d). Furthermore, the H3K27 trimethylation was downregulated in p65 knockout (p65 KO) cells but could be restored by the re-expression of p65 (Fig. 1e). To further confirm our results, we also studied the effects of NF-κB on the methylation levels of H3K27. Our findings revealed that the NF-κB could upregulate H3K27me1, -me2, and -me3 levels (Fig. 1f, g and Fig. S1e, f). Together, the above results showed that adjusting the activity of NF-κB in glioblastoma cells promotes the methylation of H3K27.

### NF-κB selectively triggers the expression of EZH2 to promote H3K27 methylation in glioblastoma

To confirm manipulating H3K27me3 status could affect glioblastoma cells, we knocked down the expression of KMKD6A/B (demethylase) and EZH2 (methyltransferase) and then detected the proliferation of glioblastoma cells (Fig. S2j-o). Our results validated the effection of H3K27me3 in glioblastoma.

**Figure 2:**
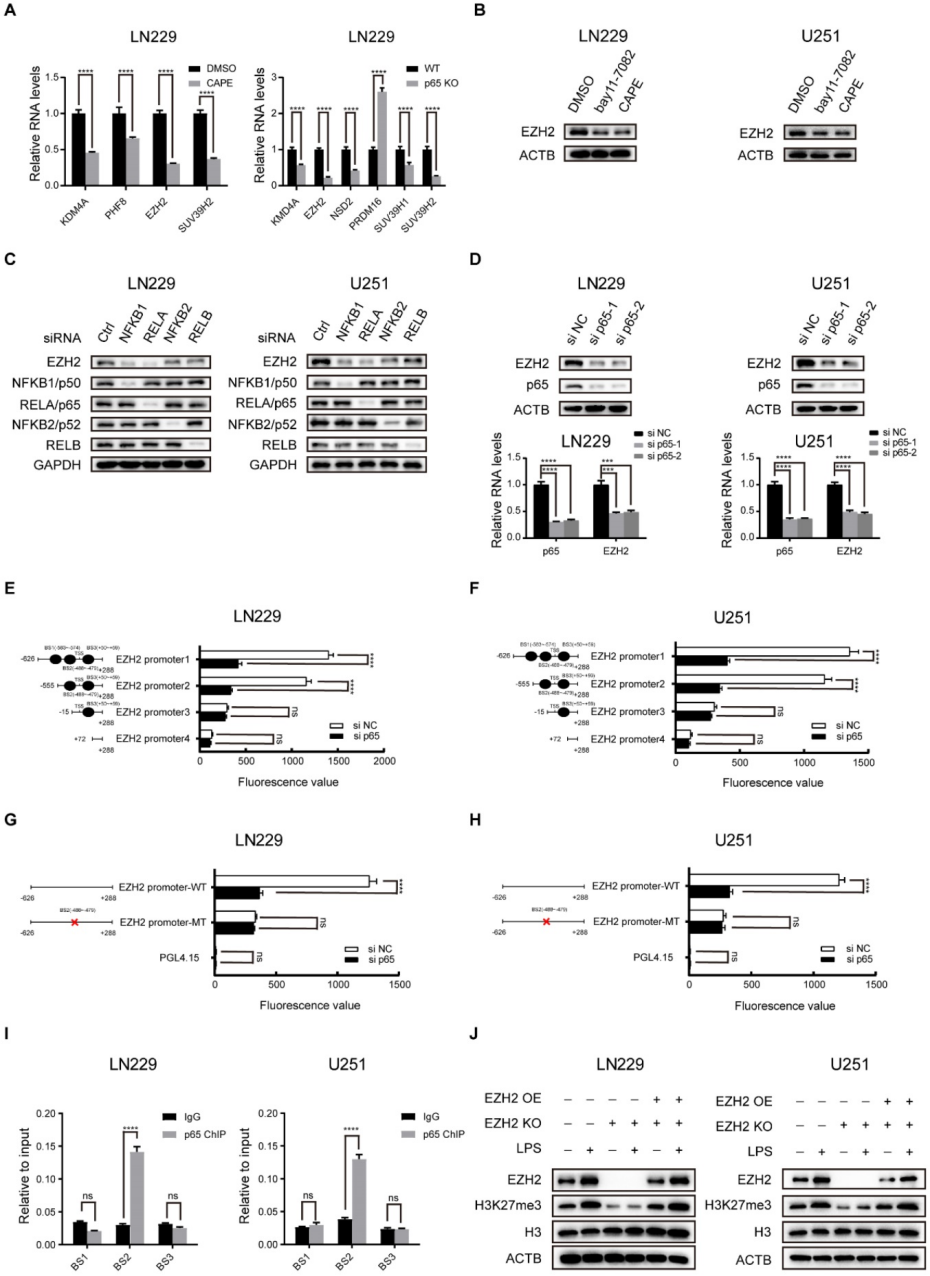
** NF-κB selectively triggers the expression of EZH2 to promote H3K27 methylation in glioblastoma. a** Relative mRNA expression of H3K27 specific methyltransferase and demethylase in LN229 and U251 cells were measured by RT-qPCR. **b** Inhibiting the activation of the NF-κB pathway by treating with 5μM bay11-7082 or 3μM CAPE and then detecting the protein expression levels of EZH2 in LN229 and U251 cells. **c** The EZH2 protein levels determined by western blot in LN229 cell lines, which was transfected with siRNAs directed against the major components of the NF-κB canonical and non-canonical pathway for 48h. **d** The expression levels of EZH2 in LN229 and U251 cells after knockdown of p65. **e,f** Luciferase activities after truncation fragments in LN229 and U251 cells.** g**,** h** Luciferase activities after mutation of -488 bp ~ -479 bp fragments in LN229 and U251 cells.** i** ChIP-qPCR of the EZH2 promoter, immunoprecipitated with antibodies against p65 in LN229 cells. **j** Western blot shows the level of EZH2 and H3K27me3 in the LN229 and U251 cell lines of EZH2 KO and EZH2 KO+OE after activating the NF-κB pathway by LPS compared to the control group. Values are expressed as the means±SD from three experiments, and the asterisk indicates the statistical significance compared to the controls (*, p < 0.05, **, p < 0.01, ***, p < 0.001, ****, p < 0.0001).

Histone methyltransferases and demethylases regulate histone methylation wildly 29. To identify the enzymes potentially responsible for H3K27 trimethylation in glioblastoma, we reviewed the list of H3K27 specific methyltransferases and demethylases from GO term (GO ID: 0046976, 0071558). Next, we used RNA-seq to identify the histone lysine methyltransferases and demethylases differentially expressed in LN229 cells following treatment with the potent NF-κB inhibitor CAPE and p65 KO (Fig. 2a and Fig. S2a, b, c). Recognized as an H3K27 specific methyltransferase, EZH2 was found to be one of the candidates. Our results revealed that EZH2 expression was modulated upon treatment with NF-κB activators and inhibitors (Fig. 2b and Fig. S2d).

Five members of the NF-κB family form several homodimers or heterodimers, but the most dominant form is p50/p65 that regulates the canonical NFκB pathway 30. To identify the exact NF-κB pathway affecting the regulation of EZH2 in glioblastoma, we used siRNAs to knockdown individual NF-κB family members and investigated EZH2 protein expression in LN229 and U251 cells (Fig. 2c). The results suggested that the NF-κB canonical signalling is the primary pathway to regulate EZH2 expression in glioblastoma. In agreement, the correlation coefficient(r) between EZH2 and p65 expression is also the highest amongst NF-κB subunits in glioblastoma tissues from the CGGA database (Fig. S2e). Consequently, we focused our study on the p65, a major subunit of the canonical pathway involved in NF-κB heterodimers.

p65 depletion or overexpression in the LN229 and U251 potently changed EZH2 expression both in protein and mRNA levels (Fig. 2d and Fig. S2g). Through analysis of ChIP-seq data from GEO (GSE55105) and motif prediction from ALGGEN, we identified that p65 could potentially bind to several regions -583bp/-574bp, -488bp/-479bp and +50bp/+59bp in the human EZH2 promoter (Fig. S2g, h). To determine the region(s) of EZH2 promoter involved in NF-κB regulation, we performed transient transfection with a series of truncated and mutated EZH2 promoter/luciferase reporter constructs in LN229 and U251 cells (Fig. 2e-h). The results revealed that p65 regulates the EZH2 promoter via the -488bp/-479bp region in glioblastoma cells. ChIP-qPCR was used to examine the endogenous occupancy of this site by p65 in LN229 and U251 cells and demonstrated enrichment of p65 specifically on this region of the EZH2 promoter (Fig. 2i), confirming NF-κB regulates EZH2 expression by transcription via its promoter. It has been proposed that EZH2 interacts with NF-κB to promote its activation 31. However, our co-IP assays did not detect the interaction between EZH2 and p65 in glioblastoma cells (Fig. S2f).

To establish the prominence of EZH2 in mediating the NF-κB-induced H3K27 trimethylation, we used LPS to activate NF-κB in WT, EZH2 KO and EZH2 KO+OE LN229 and U251 cells. The results indicate that EZH2 is crucial in inducing H3K27 trimethylation via NF-κB in glioblastoma cells (Fig. 2j).

### NF-κB inhibition triggers profound epigenetic reprogramming in glioblastoma

**Figure 3:**
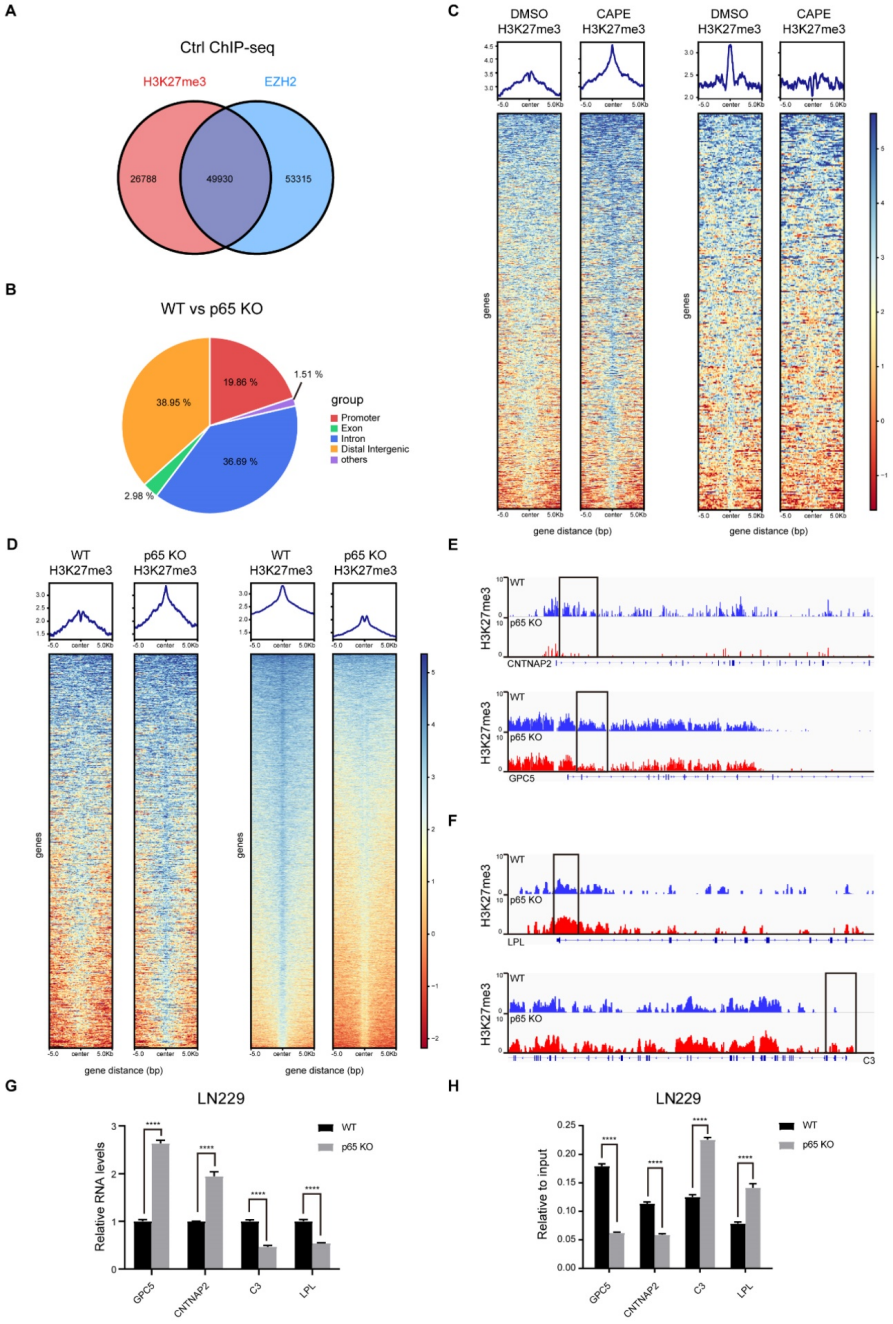
** NF-κB inhibition triggers profound epigenetic reprogramming in glioblastoma. a** Venn diagram shows the overlapping between EZH2 and H3k27me3 enrichment in control. **b** The pie chart shows the percentage of each type of differential enrichment region in WT and p65 KO LN229 cells. **c, d** Heatmap representation of regions with either loss or gain (log_10_ likely-hood ratio > 3) of H3K27me3 in p65 KO LN229 cells compared with WT. The log_2_(IP/Input) signal is plotted for each replicate, centered on the differential peak ± 5 kb. The same assay was performed in LN229 treated with DMSO or CAPE. **e-f** IGV browser views for H3K27me3 ChIP-seq libraries at four representative loci. The y axis corresponds to the ChIP-seq signal intensity. Gene representation at each locus is shown at the bottom. **g-h** RT-qPCR assay detected the differential mRNA levels analyzed by RNA-seq and the differential peaks analyzed by ChIP-seq. Values are expressed as the means±SD from three experiments, and the asterisk indicates the statistical significance compared to the controls (*, p < 0.05, **, p < 0.01, ***, p < 0.001, ****, p < 0.0001).

In glioblastoma, NF-κB activates global methylation of H3K27 through EZH2. Recent research has also established that chronic inflammation can lead to epigenetic reprogramming in macrophages 32. However, whether NF-κB regulates the redistribution of H3K27me3 remains unclarified. Thus, we performed ChIP-seq to uncover the mechanisms involved. Existing studies have shown that EZH2 is a classic methyltransferase that regulates H3K27me3. In concordance, we analysed the enrichment regions of H3K27me3 and EZH2 in the untreated controls and found that EZH2 and H3K27me3 had a total of overlapping 49,930 peaks (Fig. 3a). The top 10 pathways from enrichment analysis of the overlapping genes were presented in Fig. S3a.

Next, we explored further the influence of NF-κB on the distribution of H3K27me3. Through differential analysis, we selected peaks with a log_10_ likely-hood ratio > 3 to generate heatmaps. The experimental results showed that in the CAPE group, 733 peaks were increased and 894 peaks reduced, while in the KO group, 672 peaks were increased and 6412 peaks reduced, indicating that inhibition of NF-κB can mediate epigenetic reprogramming through H3K27me3 (Fig. 3c, d). To uncover the function of H3K27me3 redistribution on glioblastoma malignant progression, we performed GO analysis on the H3K27me3 redistributed genes and found that altered genes expression plays a critical role in promoting tumorigenesis of glioblastoma (Fig. S3d, e).

Based on the functions of genes silenced through H3K27me3 33, we decided to focus on the genes enriched in the promoter regions (Fig. 3b and Fig. S3f). There were 386 differentially enriched promoters in the inhibitor group, while the KO group had 822 differential regions. To investigate which genes are both varyingly enriched and expressed, we compared the 822 differentially enriched genes screened by ChIP-seq with the differentially expressed genes filtered by RNA-seq. The analysis showed that 133 genes with reduced enrichment had their RNA expression up-regulated, and six genes with increased enrichment had their RNA expression down-regulated upon p65 KO (Fig. S3g). We then selected the tumour suppressor genes GPC5 and CNTNAP2 and the oncogenes C3 and LPL from the down- and up-regulated expression groups respectively for further validation (Fig. 3e, f). The subsequent RT-qPCR analysis confirmed the ChIP-seq and RNA-seq results (Fig. 3g, h).

### NF-κB signalling promotes the proliferation and migration of glioblastoma is partly dependent on EZH2

**Figure 4:**
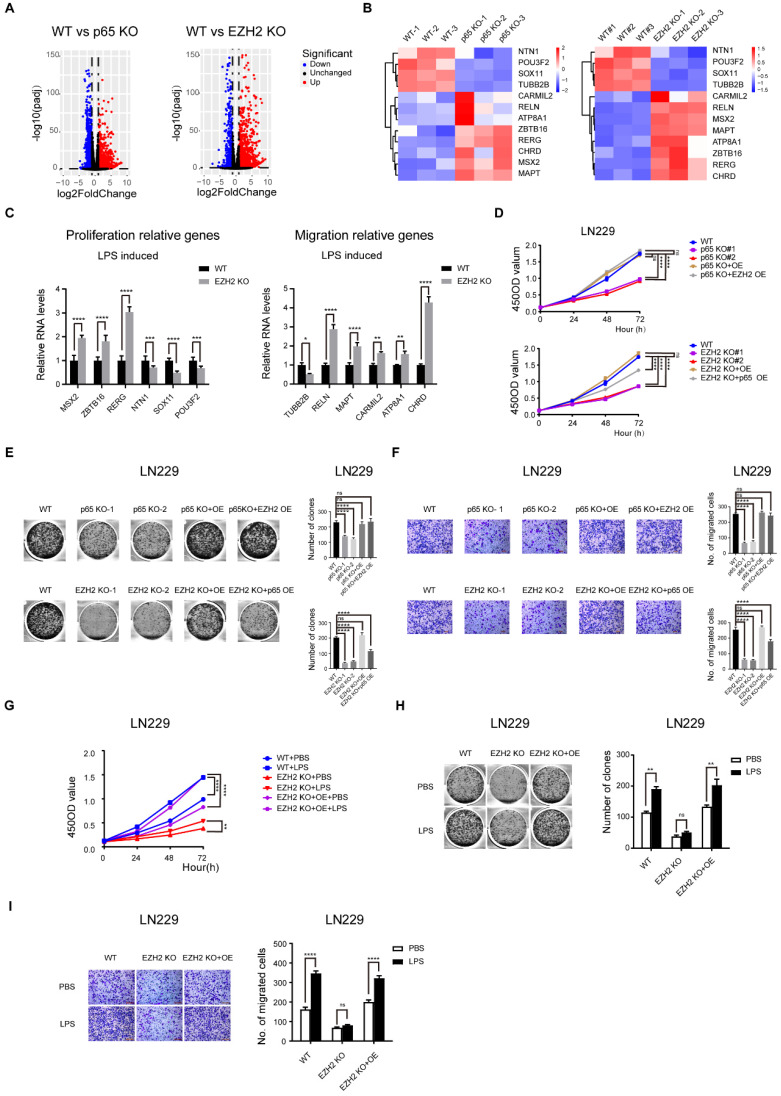
** NF-κB signalling promotes the proliferation and migration of glioblastoma is partly dependent on EZH2. a** Volcano plots illustrate differentially regulated genes expression between WT and p65/EZH2 KO LN229 cells identified by RNA-seq. Values are presented as the log_2_ of foldchange. **b** Heatmaps of candidate genes relative to proliferation and migration were both regulated by NF-κB and EZH2. **c** RT-qPCR detects the mRNA levels of candidate genes relative to proliferation and migration in WT and EZH2 KO LN229 cells treated with LPS. **d-f** CCK-8, colony formation, transwell assays in the LN229 cell line of WT, p65 KO, p65 KO with p65 ectopic overexpression and p65 KO with EZH2 ectopic overexpression groups or WT, EZH2 KO, EZH2 KO with EZH2 ectopic overexpression and EZH2 KO with p65 ectopic overexpression groups. **g-i** CCK-8, colony formation, transwell assays in WT, EZH2 KO and EZH2 KO with EZH2 ectopic overexpression LN229 cells treated with PBS or LPS. Values are expressed as the means±SD from three experiments, and the asterisk indicates the statistical significance compared to the controls (*, p < 0.05, **, p < 0.01, ***, p < 0.001, ****, p < 0.0001).

Both NF-κB and EZH2 have previously been reported to be crucial for the malignant progression of glioblastoma 14, 34. We have also demonstrated that NF-κB induces epigenetic reprogramming through EZH2. However, whether NF-κB regulates the malignant progression of glioblastoma through EZH2 is still unclear. To test this conjecture, we first used CRISPR/Cas9 to genetically delete EZH2 and p65 in glioblastoma cells (Fig. S5f-j). To identify the roles of NF-κB and EZH2 in glioblastoma gene expression, we compared the RNA-seq results of the p65 and EZH2 KO glioblastoma cells with their respective WT control cells. The volcano plot illustrated that 2043 differentially expressed genes upon p65 KO and 1961 upon EZH2 KO (Fig. 4a). We performed GO analysis of the differential genes, and the results suggested that both p65 and EZH2 regulate the malignant progression of glioblastoma cells by affecting their proliferation, apoptosis, cell cycle, and migration (Fig. S4a). Next, we tested if the tumorigenic functions of NF-κB are dependent on EZH2. To this end, we analysed the 794 p65-controlled genes whose expression is also regulated by EZH2 (708 upregulated and 88 downregulated genes, Fig. S4b), and the results showed that these genes considerably regulate glioblastoma proliferation and migration (Fig. S4c). Finally, we selected a number of core p65-regulated genes related to proliferation and migration to verify their regulation by EZH2 using RT-qPCR, suggesting that the expression levels of these p65-regulated genes are controlled by EZH2 (Fig. 4b, c).

Next, we performed CCK-8, colony formation, and transwell assays in LN229 and U251 cell lines with p65 knockout (KO), p65 KO with p65 ectopic overexpression (OE) and p65 KO with EZH2 ectopic overexpression, respectively (Fig. 4d-f and Fig. S4 d-f). It was found that ectopic overexpression of both p65 and EZH2 could rescue the deletion of p65 in p65 KO cells, suggesting NF-κB targets EZH2 to promote glioblastoma cell proliferation and migration. Conversely, p65 could only partially rescue the effects of EZH2 KO, suggesting that EZH2 functions downstream of NF-κB.

To further confirm that EZH2 plays a pivotal role in NF-κB-mediated proliferation and migration, we used LPS to activate NF-κB in LN229 and U251 cells with EZH2 KO, EZH2 KO and EZH2 ectopic expression. The glioblastoma cells were examined by CCK-8, colony formation, and transwell assays. Our results indicated that LPS, a potent activator of NF-κB, can significantly enhance proliferation and migration in control glioblastoma cells but not in EZH2-KO cells. However, this loss in oncogenic phenotypes caused by EZH2-KO could be reversed through re-expression of EZH2 (Fig. 4g-i and Fig. S4g-i). Together, our findings suggest that NF-κB promotes glioblastoma proliferation and migration via EZH2 dependent and independent pathways.

### NF-κB signalling promoting the apoptosis and cell cycle of glioblastoma is mostly independent of EZH2

**Figure 5:**
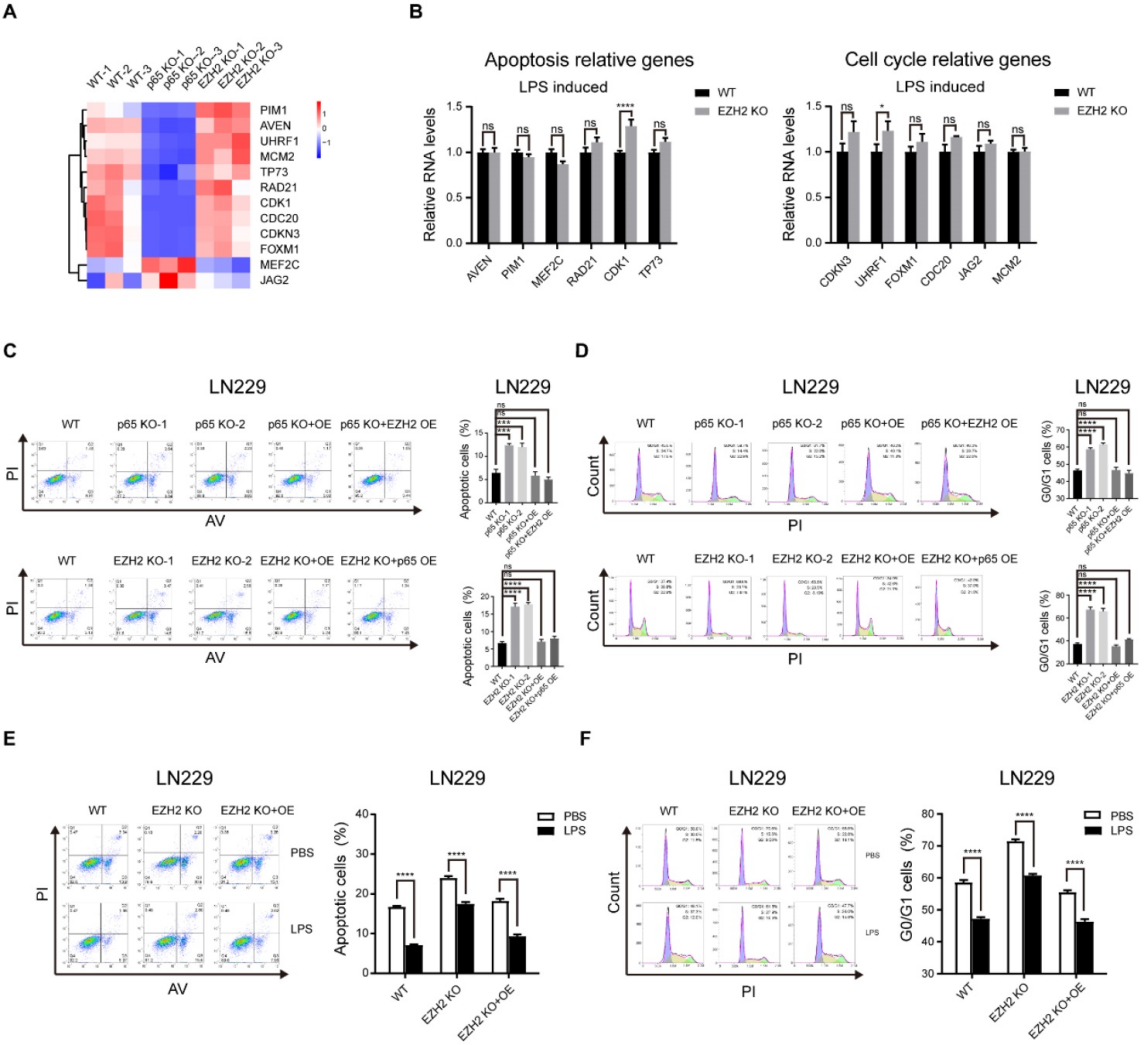
** NF-κB signalling promoting the apoptosis and cell cycle of glioblastoma is mostly independent of EZH2. a** Heatmap of candidate genes was only regulated by NF-κB. **b** RT-qPCR detects the mRNA levels of genes relative to apoptosis and cell cycle in WT and EZH2 KO LN229 cells treated with LPS. **c, d** Cell cycle and apoptosis assays in the LN229 cell line of WT, p65 KO, p65 KO with p65 ectopic overexpression and p65 KO with EZH2 ectopic overexpression groups or WT, EZH2 KO, EZH2 KO with EZH2 ectopic overexpression and EZH2 KO with p65 ectopic overexpression groups. **e, f** Cell cycle and apoptosis assays in WT, EZH2 KO and EZH2 KO with EZH2 ectopic overexpression LN229 cells treated with PBS or LPS. Values are expressed as the means±SD from three experiments, and the asterisk indicates the statistical significance compared to the controls (*, p < 0.05, **, p < 0.01, ***, p < 0.001, ****, p < 0.0001).

Next, we pursued the EZH2-independent NF-κB-regulated glioblastoma oncogenic pathways. To this end, we performed GO analysis on 1249 genes regulated only by p65 and 1167 genes controlled only by EZH2, and found that NF-κB regulates the cell cycle and apoptosis processes independent of EZH2 (Fig. S5a). Then, we selected some representative cell cycle and apoptotic genes for verification with RT-qPCR, which confirmed our findings (Fig. 5a, b). Next, we used the above groups of selected genes to perform cell cycle and apoptosis assays. The results verified that NF-κB controls the cell cycle and apoptosis processes mostly independent of EZH2 (Fig. 5c-f and Fig. 5 Sb-e, k-n).

### Glioblastoma can be molecularly stratified for risk by NF-κB and EZH2

**Figure 6:**
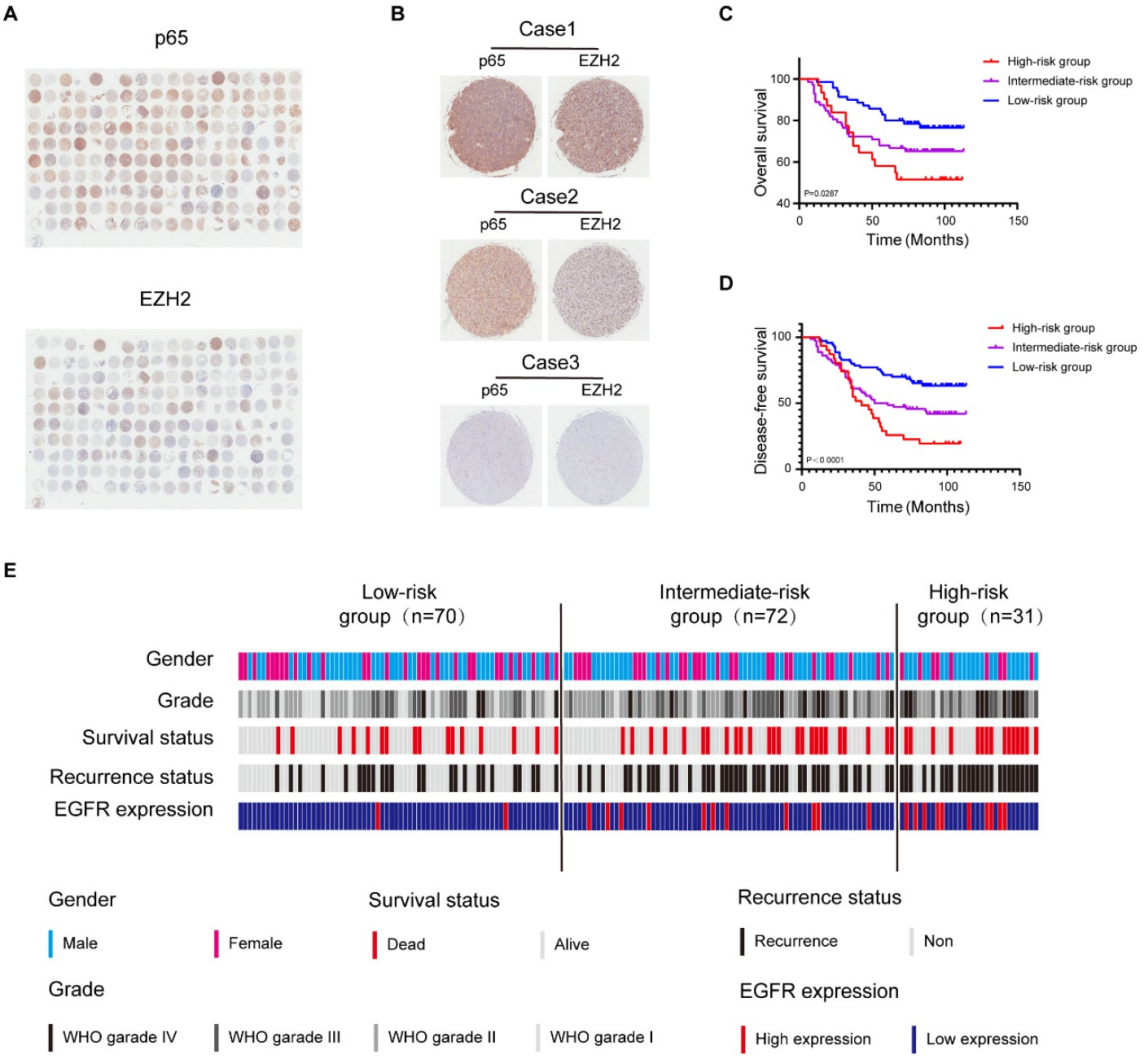
** Glioblastoma can be molecularly stratified for risk using NF-κB and EZH2. a** High and low expression of EZH2 and p65 protein in glioblastoma were detected by immunohistochemical staining Tissue Microarray(n=180). **b** Tissue Microarray EZH2 and p65 staining. Representative 40X views of tumors showing for the relative between EZH2 and p65. **c,d** The Kaplan-Meier survival curves of three risk groups. **e** Summary of clinical data and molecular characteristics of low-, intermediate and high-risk groups. glioblastoma were divided into three groups according to molecular markers. Values were expressed as the means±SD from three experiments, and the asterisk indicates the statistical significance compared to the controls (*, p < 0.05, **, p < 0.01, ***, p < 0.001, ****, p < 0.0001).

Both NF-κB and EZH2 contribute to the malignant progression of glioblastoma 14, 35, and we have demonstrated that the two oncogenes display functional interactions in glioblastoma. Initial investigations have shown that the mRNA level of EZH2 or p65 is higher in glioblastoma tissues than in normal tissues and relative to poor survival in glioblastoma patients, which we confirmed in GEPIA and CGGA database (Fig. S6a, b, l, m). However, the significance of profiling both NF-κB and EZH2 expression in combination has hitherto not been explored. To better uncover the relationship between EZH2 and p65 in the progression of human glioma, we further examined EZH2 and p65 expression in human glioma samples (n=180) by immunohistochemical (IHC) staining (Fig. 6a, b). Pearson correlation analysis indicated that EZH2 and p65 expression levels were positively correlated (Fig. S6c).

Next, we categorised the tumours (n = 173) (Supplementary Table 1) into three EZH2/p65 expression groups: low (with low p65 and EZH2 expression; n=70), intermediate (with high levels of either p65 or EZH2; n = 72), and high-(with high levels of either p65 or EZH2;n = 31). The statistical analysis illustrated that the EZH2/p65 axis was dramatically relative to age, glioma grades and EGFR expression, suggesting that high levels of both p65 and EZH2 expression are correlated to a higher risk of glioblastoma development and progression (Supplementary Table 2). In agreement, Kaplan-Meier analysis also indicated that high EZH2/p65 expression is associated with a poorer prognosis (Fig. 6c-e). Statistic of 5-OS and 5-DFS on different clinicopathological factors by Kaplan-Meier shown the details (Fig. S6d-k and Supplementary Table 3).

### Synergistic effect induced by inhibition of EZH2 and NF-κB in glioblastoma

Recent research has also demonstrated that inhibiting EZH2 methyltransferase activity in cancer cells is synthetic lethal with deficiency of the chromatin remodeling gene ARID1A 36. Our results thus far identified that the development of glioblastoma is regulated by NF-κB through EZH2-dependent and independent pathway and led us to hypothesize that targeting of NF-κB can enhance the anti-cancer effects of EZH2 inhibition in glioblastoma.

**Figure 7:**
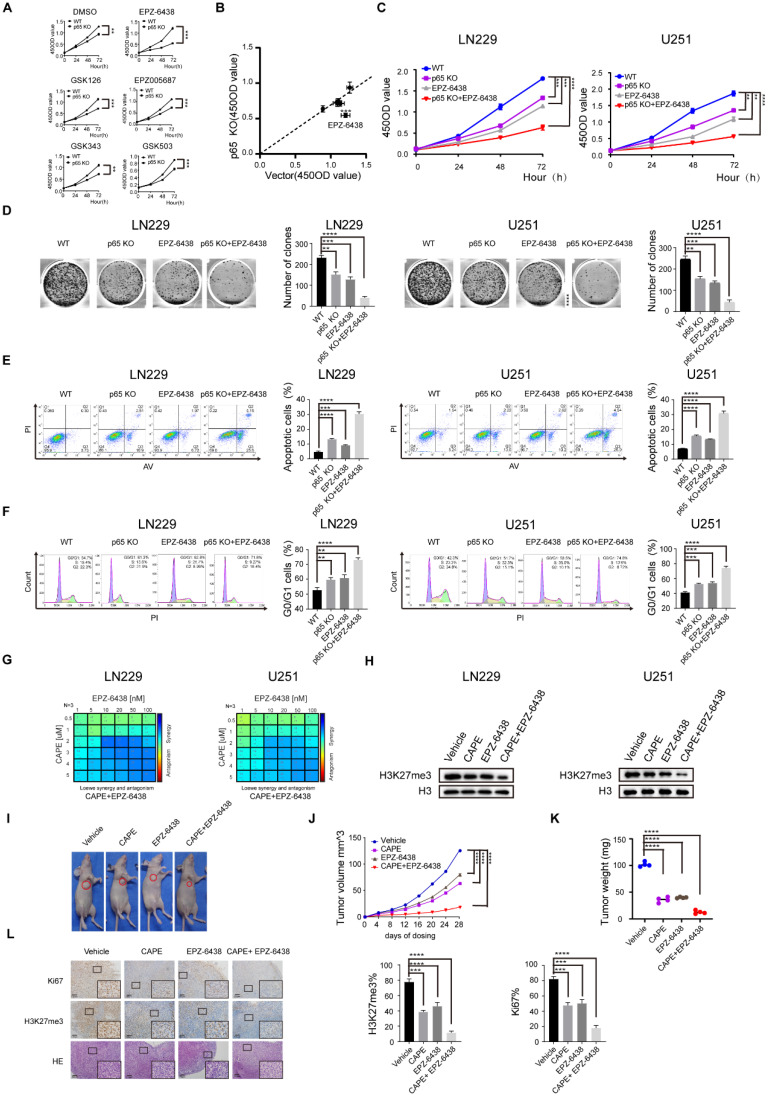
**Synergistic effect induced by inhibition of EZH2 and NF-κB in glioblastoma. a** WT and p65 KO LN229 cells were treated with DMSO or EZH2 inhibitors, including EPZ-6438, GSK126, EPZ005687, GSK343, GSK503. Cell viability was measured at different time points after treatment using CCK8 assays. **b** The x-axis indicates the 450OD value formed by treated WT LN229 cells, and the y-axis shows the 450OD value by p65 KO LN229 cells treated with the same small molecule. **c** The EZH2 small molecule inhibitor EPZ-6438 sensitizes p65-KO LN229 and U251 cells detected by CCK8 assay. **d~f** Colony formation, cell cycle and apoptosis assay were performed in the sample groups. **g** LN229 and U251 cell lines were treated with increasing doses of EPZ-6438 combined with CAPE for 24 hours, and proliferation was monitored by CCK8-kit. LOEWE scores and heatmaps were calculated by Combenefit. **h** LN229 and U251 cells were harvested from drug-therapeutic cells at the end of the treatment (24h), then immunoblotted as indicated. **i** Subcutaneously implanted mice were treated with EPZ-6438, CAPE or combination. Images of representative tumors for every group at day 28. **j, k** Changes of tumor volume(j) and tumor weight(k) treated with CAPE, EPZ-2438 or combination groups during day 28. **l** IHC staining using Hematoxylin and eosin (H&E) for Ki67 and H3K27me3. Values are expressed as the means±SD from three experiments, and the asterisk indicates the statistical significance compared to the controls (*, p < 0.05, **, p < 0.01, ***, p < 0.001, ****, p < 0.0001).

Firstly, we estimated five small molecule EZH2 inhibitors for their efficacy in restricting the growth of WT and p65 KO glioblastoma cells using the previously established inhibitor IC50 values (Fig. 7a). The results indicated that all five small molecule inhibitors significantly and specifically suppressed the proliferation of p65 KO cells contrasted to WT control cells (Fig. 7b), with EPZ-6438 displaying the highest selective efficiencies against p65 KO cells. We further demonstrated that p65 KO or pharmacological inhibition of EZH2 alone was insufficient to limit LN229 and U251 cell proliferation in both short-term CCK-8 and long-term colony formation assays *in vitro*. Results also showed that cancer cell proliferation was significantly attenuated when NF-κB and EZH2 inhibition were combined (Fig. 7c, d). This effect was also confirmed in cell cycle and apoptosis assays (Fig. 7e, f). Concisely, the above outcomes showed that inhibition of both NF-κB and EZH2 is synthetic lethal in glioblastoma.

Despite being tested in clinical studies, some of the EZH2 inhibitors have been reported to be involved in developing multidrug resistance. However, the anti-proliferative effects of EPZ-6438 have been shown to be significantly enhanced when combined with the chemotherapeutic regime CHOP (cyclophosphamide, doxorubicin, vincristine, and prednisone), suggesting EZH2 inhibitors have potent anticancer activities when administered with other therapeutics 37, 38. Our results have shown that NF-κB depletion sensitizes EZH2 inhibitors to reducing glioblastoma cell viability, an observation that has not been described previously. Given the existence of NF-κB inhibitors and considering our findings, we speculated that such NF-κB inhibitors could synergize with EPZ-6438 to reduce the growth of glioblastoma cells.

The proliferation results indicated that the EZH2 inhibitor EPZ-6438 synergised with the NF-κb inhibitor CAPE in inducing anti-proliferative activities in both LN229 and U251 glioblastoma cell lines (Fig. 7g and Fig. S7a, b). Consistently, western blot analysis also showed that combining CAPE with EPZ-6438 had a more significant effect on repressing H3K27me3 than either agent alone in LN229 and U251 cells (Fig. 7h). Similar enhanced anti-proliferative functions were also observed when combining CAPE with EPZ-6438 in colony formation, cell cycle, apoptosis assays (Fig. S7c-e). To corroborate these findings, we evaluated the combinational effects of CAPE and EPZ-6438 in glioblastoma BAlB/c-mouse xenograft models (Fig. 7i and Fig. S7f). Although CAPE or EPZ-6438 alone effectively restricted glioblastoma tumour growth, the combination more effectively blocked tumour progression than the single-agent alone *in vivo* (Fig. 7j, k). Lastly, we used IHC to examine the expression levels of KI67 and H3K27me3 and HE staining to detect the progression of the tumours (Fig. 7l). We found that simultaneous inhibition of CAPE and EPZ-6438 suppresses glioblastoma growth more effectively than either agent alone *in vivo*.

Together, these data demonstrated that NF-κB inhibitor CAPE synergises with the EZH2 inhibitor EPZ-6438 to suppress the development of glioblastoma both *in vitro* and *in vivo*.

## Discussion

Glioblastoma is still an incurable disease and lacks any appropriate targets for effective treatment in the clinic. The inflammatory microenvironment is an essential component of the vast majority of tumours through enhancing their malignant progression 7, 8. The blood-brain barrier is destroyed by glioblastoma, causing chronic neuroinflammation that is a cardinal feature of glioblastoma biology 3. Chronic inflammation is relative to poor outcomes and reduced responsiveness to treatment in tumour (including glioblastoma) patients 7, 39. At the same time, epigenetic modifications have also been described to be essential in tumorigenesis, and are therefore exploited as therapeutic targets for treating cancer 40. Recent evidence indicates inflammation is associated with epigenetic modifications, which is supported by the observation in machrophages 32. However, their functional connection and combinatory impact on cancer, especially on glioblastoma, remain unclear. Therefore, exploring the potential link of inflammation to epigenetic alterations can unveil novel and effective strategies for the stratification and treatment of glioblastoma.

NF-κB serves as a significant link between chronic inflammation and cancer 11. Like many other malignancies, glioblastoma has been demonstrated to possess high levels of NFκB activity 27. Histone lysine methylation is one of the most important epigenetic modifications, playing a critical role in health and disease 18. Here, we found that NF-κB activation specifically dictates H3K27 methylation via upregulating transcription of EZH2 which is also critical for promoting glioblastoma malignant progression, thus revealing the crosstalk between inflammation and epigenetics. To this end, we firstly detected the global expression of H3K4me3, H3K9me3, H3K27me3, and H3K36me3. We found that NF-κB only modulates the methylation levels of H3K27, but not H3K4, H3K9 and H3K36 in glioblastoma cells. To further identify histone enzymes involved in the methylation of H3K27, we screened a number of methyltransferases and demethylases in glioblastoma cells and identified EZH2 as the principal methyltransferase specifically responsible for H3K27 trimethylation. Mechanistically, we confirmed that the canonical NFκB signalling mediated by p65/p50 directly regulates the transcription of EZH2, resulting in a global change of expression and distribution of H3K27me3. In agreement, NF-κB2, the regulator of the non-canonical NFκB signalling, has previously been found to activate EZH2 to promote senescence bypass in melanoma 41. In glioblastoma, however, it is the canonical NFκB signalling (rather than the non-canonical) that is constituvely activated to drive EZH2 expression and tumour progression. Meanwhile, our findings are opposed to the likelihood that NFκB physically binds with EZH2 to regulate the activity of the latter molecule as reported by others 31. Taken together, we identify a key axis from the canonical NF-κB pathway to the EZH2 signaling, linking chronic inflammation and epigenetic reprogramming and promoting the malignant progression of glioblastoma.

Based on genetic and epigenetic features, WHO has changed the CNS Tumour Classification to a molecular categorization such as IDH-wildtype and IDH-mutant glioblastoma, H3K27M-mutant diffuse midline glioma, and p65 fusion-positive ependymoma, etc. 43, 44. Meanwhile, these aberrant molecules also represent attractive therapeutic targets for drug development. Thus far, glioblastoma is still a fatal disease without efficient therapeutic treatment. Consequently, it becomes particularly urgent to search for novel molecular biomarkers to predict the prognosis and to make individualized regimens. EZH2 or NF-κB over-activation has previously been determined relative to the development of glioblastoma 14, 34. The prognostic significance of their combination has hitherto been uncovered. It is the first time that we verified co-activation of NF-κB and EZH2 confers the poorest clinic outcome and the risk and prognosis of glioblastoma can be reliably stratified according to the expression of EZH2 and p65 [low-risk: low p65 and EZH2 expression; intermediate-risk: high either p65 or EZH2 expression; and high-risk: high p65 and EZH2 expression; DFS and OS (p < 0.0001)]. This novel strategy will aid the diagnosis and clinical management of patients with glioblastoma. Moreover, these findings prove further clinical evidence for the idea that EZH2 and NF-κB combine to promote the development and progression of glioblastoma.

EZH2 is a crucial subunit of the PRC2 and catalyzes H3K27me2/3, which are linked to transcriptional silence 23, 45. In multiple cancers, EZH2 is frequently over-activated to promote tumorigenesis, making it a promising drug target for cancer treatment 46. Whereas the efficacy of EZH2 inhibitors (EZH2i) as single agents has not lived up to their earlier high expectations in the clinic. Accordingly, EZH2i treatment alone is ineffective or causes multidrug resistance in some EZH2-overexpressing solid tumours. Nevertheless, further preclinical studies also suggest that combining EZH2i with other therapies has a synergistic anti-cancer impact, revealing cancer cells can be sensitised to EZH2i in the presence of an additional perturbation 47.

Being an essential transcription factor, NF-κB is implicated in regulating hundreds of genes in various types of biological processes and diseases 48. As mentioned above, NF-κB is a lynchpin linking chronic inflammation and cancer, and it is frequently activated in multiple cancers. Just like EZH2, NF-κB has long been regarded as a potential therapeutic target of cancer. However, inhibition of NF-κB alone will not affect most solid tumours 49. Paradoxically, NF-κB inhibition alone may promote tumour progression due to the pro-apoptotic potential of NF-κB 50. In past decades, no any encouraging results have been acquired in all clinical trials except that various inhibitors, including natural compounds like CAPE are guaranteed in safety and availability 51. Therefore, it is still a great challenge to targeting of NF-κB for cancer treatment. It is notable that NF-κB may regulate epigenetic reprogramming and biological processes via EZH2-dependent and EZH2-independent manner, suggesting that NF-κB can potentially cooperate with EZH2 in more than one way to impact tumorigenesis. In this study, cancer cells can be induced to a Synergistic effect by EZH2i upon genetic ablation of NF-κB, indicating that redundant survival pathway(s)/mechanism(s) of EZH2 can compensate for the loss of NF-κB activity. EPZ-6438 is one of the most effective EZH2i and is performed Phase I clinical trial in several types of cancer patients. Our study demonstrated that combined targeting of NF-κB and EZH2 with CAPE and EPZ-6438 respectively have synergistic suppression of cancer *in vitro* and *in vivo* and therefore provides information for devising strategies of Synergistic effect for the treatment of glioblastoma. However, whether this pharmacological effect also holds in other cancers requires further investigation.

The exact underlying mechanistic basis for the cooperation between the NF-κB and EZH2 inhibitors remains unclear and warrants further investigation. Possibly, it is as a result of NF-κB could induce EZH2 independent and dependent epigenetic reprogramming via H3K27me3. In concordance with this idea, a recent study has shown that genetic ablation/deletion or pharmacological inhibition of EZH2 triggered feedback activation of NF-κB via the EZH2-SOX9-TNFRSF11A signalling axis in prostate cancer cells. Mechanistically, EZH2 represses SOX9 gene expression and inactivation causes derepression of SOX9, a transcriptional regulator of TNFRSF11A, which in turn is a receptor activator of NF-κB 52. In addition, EZH2 has also been demonstrated to bind to NKILA and disrupt the reciprocal negative feedback inhibition between NKILA and NF‐κB 53.

## Materials and methods

### Mice

Nud mice were reared in SPF. On the first day, we implanted 5×10^6^ LN229 cells in 200 µl PBS into mice subcutaneously. The mice were then randomly apart into four groups. Tumour volume was measured per 4 days. We intraperitoneally injected CAPE (Selleck, S7414, 10 mg/kg in DMSO) or orally administered EPZ-6438 (Selleck, S7128, 100 mg/kg in corn oil) to mice for 28 consecutive days. Twenty-eight days after injection, we euthanized the mice and gauged the tumour weight. The tumours were examined by H&E or IHC staining following the protocol in the kit. The animal assays were conducted in obedience to the recommendations from the animal ethics committee (Dalian Medical University) and conformed to local regulations.

### Tissue microarrays

Tissue microarray slides containing 180 glioblastoma were handled as follows: The slides were deparaffinized in xylene by heating for 1 hour at 60 °C and subsequent rehydration with ethanol. Antigen retrieval was performed in a chamber containing citrate buffer (pH 6.0) for 20 min maintaining at a sub-boiling temperature. Endogenous peroxidase activity was blocked by treating the slides in methanol with 3% hydrogen peroxide for 10 min. Samples were blocked with 10% goat serum for one h each at room temperature. 200 μl primary antibody was added to each slide and incubated overnight at 4 °C. The second antibody with Streptavidin/Peroxidase was added for immunochemistry staining assay. For each tissue sample, protein expression was scored according to the staining color: negative staining (no yellow); low staining (light yellow); moderate or high staining (yellow-brown or brown). With prior written consent from patients, all the tissue samples had been obtained before anti-cancer treatment. All of the methods in this study were in accordance with the approved guidelines, and all of the experimental protocols were approved by the ethics committee of Xinchao (Shanghai).

### Cell Culture

293T cells were cultivated in DMEM with 1% penicillin (100 IU/ml) and streptomycin (100 mg/ml) and 10% fetal bovine serum (FBS). LN229 and U251 (glioblastoma cell lines) were nurtured in DMEM medium supplemented with 5% or 10% FBS. All cells were bought from American Type Culture Collection (ATCC) and continuously cultured at 37 °C with 5% CO2 incubator. Cell lines were routinely confirmed by STR allele profile and validated to be free of mycoplasma via the Lonza kit (LT07-218; Lonza Biologics, Guangzhou, China).

### Immunofluorescence

Five ×10^4^ LN229 and U251 cells were grown on coverslips, which were cultured for one night. After fixing in 4% paraformaldehyde for 15 min, cells were washed three times and permeabilized for 15 min. Cells were blocked before incubating with a certain primary antibody in a cold room overnight. After 12 h, cells were washed three times in PBS before incubation for 1 h with a corresponding fluorescent secondary antibody, followed by incubating with Hoechst 33342 for 15 min. Immunofluorescence images were taken by Leica SP5 Confocal Microscope (Leica Microsystems, Mannheim, Germany).

### Western Blot Analysis

Cells were collected and boiled for 10 min in the RIPA buffer (Sigma). 20 μg protein were resolved on SDS-PAGE gels and electro-transferred onto nitrocellulose membranes (Millipore, Merck, Shanghai, China) using a power supply (BIO-RAD, Guangzhou, China) at 250 mA for two h. Membranes were blocked by non-fat milk. Antibodies: p65 (Cell Signalling Technology, Shanghai, China; 6956, 1:1000), p-p65 (Ser536, Cell Signalling Technology, 3033, 1:1000), EZH2 (Cell Signalling Technology, 5246 , 1:1000), H3K4me3 (Cell Signalling Technology, 9751, 1:1000), H3K9me3 (Cell Signalling Technology, 13969; 1:1000), H3K36me3 (Cell Signalling Technology, 4909, 1:1000), H3K27me3 (Cell Signalling Technology, 9733, 1:1000), H3K27me2 (Cell Signalling Technology, 9728, 1:1000), H3K27me1 (Cell Signalling Technology, 84932, 1:1000), H3 (Abcam, Hangzhou, China; Ab1791, 1:10,00), ACTB(Cell Signalling Technology, 4970, 1:1000), NFKB1(Cell Signalling Technology, 13586, 1:1000), NFKB2 (Cell Signalling Technology, 37359, 1:1000) and GAPDH (Cell Signalling Technology, 97166, 1:3000). The blots were covered with respective primary antibodies at cold room and corresponding secondary antibodies (1:3000, ThermoFisher, USA). The membranes were processed using an ECL kit (ThermoFisher, USA).

### RNA extraction and qRT‐PCR

The total cellular RNA in the glioblastoma cells was isolated by TRIZOL reagent (Invitrogen). Then, cDNA was reversed from 1µg total RNA by the RT Reagent Kit (RR037A, TAKARA, Beijing, China). The primer sequences are filed in Supplementary Table 4. Program conditions for RT-qPCR reaction are in obedience to the recommendations. For making sure authenticity, each assay was performed at least three times and normalized to the internal control, β-Actin. We used the delta CT method to calculate the gene expression levels between different groups.

### Transfection

siRNA was transfected into glioblastoma cells with lipo2000 (ThermoFisher, USA) for 48h. SgRNA was transfected into HEK293T cells along with psPAX2 (Addgene, 8454) and PMD2.G (Addgene, 12260). Viruses were harvested 48 and 72 h after transfection, which were filtered using a 0.45 μm nitrocellulose filter. Such stocks were then concentrated by ultracentrifugation or precipitation using polyethylene glycol 6000, resulting in viral titers 1×10^8^ transducing units per milliliter or above by lentivirus qPCR Titration kit (TransLvTM; TransGen Biotech Co, DongShen, China) analysis. Virus infects target cells with polybrene (8 mg/ml) after spinoculation at 4500 × g for 30 min at 4 °C. The next day, cells were subsequently screened with puromycin for 48 h (2-5 μg/ml, Sigma). After that, cells were transferred to 10 cm dishes and maintained for experimental purposes. The method of constructing an *EZH2*-coding plasmid that reported stable overexpression strain is the same as described above.

### CRISPR-Cas9

EZH2 or p65 knockout glioblastoma cells were generated by the CRISPR/Cas9 system from Zhang laboratory. In summary, *EZH2* or p65-specific sgRNAs were designed by the CRISPR design tool and constructed into the lentiCRISPR V2 vector (Addgene, MA, USA, 63592). Next, HEK293T cells were treated with the sgRNA, pVSVg and psPAX2. The viruses were concentrated by ultracentrifugation or precipitation using polyethylene glycol 6000. After spinoculation at 4500 × g for 30 min at 4 °C, cells were infected with lentiviruses (multiplicity of infection of 5 and 8μg/ml polybrene) harboring a target plasmid or empty vector control. Cells were subsequently screened with puromycin (2-5 μg/ml, Sigma) for two days. The final concentration was 60 cells per 10 ml by limiting dilution method. Then the 96-well plates were plated and cultured for seven days until every single cell grew into a monoclonal cell line. Cell clones with EZH2/p65 stable knockout were verified by RT-PCR and sequencing.

### Cell proliferation assay

Cell proliferative ability was detected using a CCK-8 kit (Merck) according to the protocol. After treatment, 1-2 × 10^3^ cells were cultivated for four days, and 10 μl of the CCK-8 kit was added. Thereafter, the samples were incubated for two hours in the dark at 37 °C with 5% CO2. 450 nm OD value was detected with a microplate reader. As for the Colony formation assay, cells (500 or 1000 cells) were cultured with a complete medium for 7 to 14 days. Cells were then washed with PBS, and fixed for 30 min and stained with 0.1% crystal violet solution.

### Flow cytometry

LN229 and U251 cells were counted (1×10^6^ cells) and treated or not treated with VP16 for 24 h at 10 μM for apoptosis assay. The Apoptosis Kit (BD Biosciences, Shanghai, China) was in obedience to recommendations. Cells were re-suspended in 500 ul 1 × Binding. We incubate 5 μl Annexin V for 15 min and 5 μl propidium iodide for another 5 min on ice. About cell cycle analysis, we washed the treated cells with PBS and incubated them with 70% ethanol at -4 °C overnight. Then we re-suspended the cells and stained them in 500 ul of PI containing 25 ug/ml RNase for 15 min at RT. Finally, the cells were performed on BD C6 (BD Biosciences) and analysed using Flowjo (SRI, USA).

### Transwell assay

LN229 and U251 were re-suspended in serum-free FBS DMEM. 5×10^3^ or 1×10^4^ cells were placed on each transwell chamber (8 μm, BD Biosciences) and the complete medium was applied under the chambers subsequently. After two days, the upper migrated cells on the membrane surface were washed with PBS, fixed for 30 min and stained with 0.1% crystal violet solution. Cells were observed under a microscope in five random fields.

### Dual-luciferase reporter gene assay

The transfected cells were subjected to luciferase assays using the Dual-luciferase Reporter Assay System (Promega, Madison, WI, USA). Promoter constructs were generated by cloning the region of the human *EZH2* promoter from -626 to +288 and inserting it between the EcoRI and KpnI restriction sites of the pGL4.15 vector (Promega). Glioblastoma cells were transfected with either the pGL4.15 (Promega) or *EZH2* promoter-luciferase constructs. After 24 h, the transfected cells were lysed and processed according to the Dual-luciferase Reporter Assay System protocol. The promoter reporter activities were detected by a microplate reader (Tecan, Switzerland) and normalized with Renilla luciferase activities.

### RNA-seq

Control and treated samples were collected using TRIZOL reaction solution (ThermoFisher). Library preparation and RNA sequencing were completed at Novagene (Beijing, China). The statistical difference in gene expression was determined by the adjusted P-value cutoff value of 0.05 and the fold change cutoff value of 2. To determine statistically significant enrichment, we use the GO-seq R package to perform gene ontology (GO) analysis of differentially expressed genes. The corrected P-value cutoff value is 0.05 at this time.

### ChIP and ChIP-seq

Cells from two independent experiments were fixed at RT for 15 min before neutralizing by glycine. The lysates were shear to approximately 200 ~ 1000 bp fragments in sonication buffer. The chromatin solutions were precipitated 4h with certain antibodies or IgG. The Immunoprecipitation was caught by magnetic beads. After the immunocomplexes were washed 5 min for three times, the captured DNA fragments were eluted by proteinase K digestion. Multiplexed libraries were sequenced either on an Illumina NextSeq 500 or a HiSeq-2500 sequencer. Peak calling for individual IPs was performed using the call peak function from the model-based analysis of ChIP-seq 2 (MACS2) program. Peaks were re-called using the pooled IP and the corresponding pooled Input with the nonmodel option. To ensure the significant (log10 likelihood ratio > 3) differential peaks, the fold enrichment of (Pooled IP/Pooled Input) was calculated for each group and normalized to reads per million. Visualized all peaks using integral Genomics Viewer (IGV) and adaptive data range settings, respectively.

### Statistical analysis

Statistical analyses were processed by Prism GraphPad. The data were presented as the mean values ± standard deviation. Each quantitative assay in this study was performed in at least independent triplicates. P < 0.05 means statistical difference.

### Database analyses

The data of differential analysis between glioblastoma and normal tissues were downloaded from GEPIA database (the glioblastoma data is from TCGA database, the normal data is from GTEx database). We downloaded RNA-seq data for 325 glioma patients with clinical data from CGGA database. First of all, we divided the samples into three panels in accordance with the expression levels of EZH2 and p65. Survival data by the Kaplan-Meier survival analysis includes life status, days of death and other variables. The relationship between EZH2 mRNA expression and NF-κB subunits mRNA expression was analysed using the Pearson correlation analysis.

## Supplementary Material

Supplementary figures and tables.
