# Supplementary material for: Chronic Inflammation Pathway NF-κB Cooperates with Epigenetic Reprogramming to Drive the Malignant Progression of Glioblastoma
Source: Int J Biol Sci. 2022 Sep 21;18(15):5770–86. doi: 10.7150/ijbs.73749 (PMC9576505; doi:10.7150/ijbs.73749)
Supplement: Supplementary file 1 — Supplementary figures and tables. [file ijbsv18p5770s1.pdf]

Supplementary figure legends

Fig. S1 Related to Fig. 1, canonical NF-κB signalling specifically activates global methylation of H3K27 in glioblastoma. **a** Cells were co-transfected with pGL4.32[luc2P/NF-kB-RE/Hygro], using pRL-TK-Renilla as an internal control in LN229 and U251 cells. After 24 hours of LPS (0.1/1/10μg/mL) or TNF-α(1/10/50ng/mL) treatment. The luciferase activity was measured and standardized to Renilla luciferase. **b** Cells were co-transfected with pGL4.32[luc2P/NF-kB-RE/Hygro], using pRL-TK-Renilla as an internal control in LN229 and U251 cells. After 24 hours of BAY11 (1/2/5μM) or CAPE (1/2/5μM) treatment. The luciferase activity was measured and normalized to Renilla luciferase. **c** After 24 hours of LPS (1μg/mL) or TNF-α(10ng/mL) treatment, levels of histone lysine methylation were detected by western blotting. **d** The expression levels of histone lysine methylation upon overexpression p65 in LN229 and U251 cells. **e**, **f** The expression levels of H3K27me1/2/3 upon overexpression p65 or NF-κB activators in LN229 and U251 cells. **g, h** the EC50 curves of two NF-κB inhibitors. Values are expressed as the means±SD from three experiments, and the asterisk indicates the statistical significance compared to the controls (*, $p < 0.05$, **, $p < 0.01$, ***, $p < 0.001$, ****, $p < 0.0001$).

Fig. S2 Related to Fig. 2, NF-κB selectively triggers the expression of EZH2 to promote H3K27 methylation in glioblastoma**. a** Volcano plots illustrated differentially regulated genes expression from RNA-seq analysis between WT and p65 KO LN229 cells and identified by RNA-seq. The same assay was performed in LN229 treated with DMSO or CAPE. Values are presented as the log_2 of foldchange. **b** Heatmaps of H3K27 specific methyltransferase and demethylase differentially expressed between DMSO and CAPE or WT and p65 KO in LN229 cells identified by RNA-seq. **d** To activate the NF-κB pathway by treating with 1ug/ml LPS or 10ng/ml TNFα and then detect the protein expression levels of EZH2 in LN229 and U251 cells. **e** Pearson correlation coefficient (r) and p-value (p) between EZH2 and NF-κB subunits expression (in log_2). **f** IP analysis of the interaction between EZH2 and p65 in LN229 and U251 cells **g** Expression levels of EZH2 in LN229 and U251 cells after overexpression of p65. **h** Enrichment of p65 in the EZH2 promoter region was detected by ChIP-seq data from GEO. **i** p65 motif in EZH2 promoter prediction from ALGGEN. **j** The expression levels of H3K27me3 in LN229 and U251 cells after knockdown of EZH2. **k, l** CCK-8 and colony formation assays were performed in LN229 and U251 cells after knockdown of EZH2. **m** The expression levels of H3K27me3 in LN229 and U251 cells after knockdown of KDM6A and KDM6B. **n, o** CCK-8 and colony formation assays were performed in LN229 and U251 cells after knockdown of KDM6A and KDM6B. Values are expressed as the means±SD from three experiments, and the asterisk indicates the statistical significance compared to the controls (*, $p < 0.05$, **, $p < 0.01$, ***, $p < 0.001$, ****, $p < 0.0001$).

Fig. S3 Related to Fig. 3, NF-κB inhibition triggers profound epigenetic reprogramming in glioblastoma. **a** GO pathway analysis of differential genes by overlapping EZH2 and H3K27me3 enrichment foldchange. **b-c** Heatmap representation of regions with either loss or gain (log_{10} likely-hood ratio > 3) of EZH2 in p65 KO LN229 cells compared with WT. The log_2(IP/Input) signal is plotted for each replicate, centered on the differential peak ± 5 kb. Each column is representative of an individual donor. The same assay was performed in LN229 treated with DMSO or CAPE. **d-e** GO pathway analysis of differential genes from CHIP-seq analysis between WT and p65 KO LN229

cells. The same assay was performed in LN229 treated with DMSO or CAPE. **f** The pie chart shows the percentage of each type of differential enrichment region in LN229 cells treated with DMSO or CAPE. **g** Venn diagrams showing the overlapping between ChIP-seq and RNA-seq. Values are expressed as the means±SD from three experiments.

Fig. S4 Related to Fig. 4, NF-κB signalling which promotes the proliferation and migration of glioblastoma is partly dependent on EZH2. **a** GO pathway analysis illustrates differentially regulated genes expression between WT and p65/EZH2 KO LN229 cells identified by RNA-seq. **b** Venn diagram shows the overlapping pathway about differential genes with EZH2 KO and p65 KO. **c** GO pathway analysis of genes both regulated by NF-κB and EZH2. **d-f** CCK-8, colony formation, transwell assays in the U251 cell line of WT, p65 KO, p65 KO with p65 ectopic overexpression and p65 KO with EZH2 ectopic overexpression group or WT, EZH2 KO, EZH2 KO with EZH2 ectopic overexpression and EZH2 KO with p65 ectopic overexpression group. **g-i** CCK-8, colony formation, transwell assays in WT, EZH2 KO and EZH2 KO with EZH2 ectopic overexpression U251 cells treated with PBS or LPS. Values are expressed as the means±SD from three experiments, and the asterisk indicates the statistical significance compared to the controls (*, $p < 0.05$, **, $p < 0.01$, ***, $p < 0.001$, ****, $p < 0.0001$).

Fig. S5 Related to Fig. 5, NF-κB signalling promoting the apoptosis and cell cycle of glioblastoma is mostly independent of EZH2. **a** GO pathway analysis of genes only regulated by NF-κB or EZH2. **b, c** Cell cycle and apoptosis assays in the U251 cell line of WT, p65 KO, p65 KO with p65 ectopic overexpression and p65 KO with EZH2 ectopic overexpression groups or WT, EZH2 KO, EZH2 KO with EZH2 ectopic overexpression and EZH2 KO with p65 ectopic overexpression groups. **d, e** Cell cycle and apoptosis assays in WT, EZH2 KO and EZH2 KO with EZH2 ectopic overexpression U251 cells treated with PBS or LPS. **f** A schematic map of the vector cassette. **g** A procedure of selection and identification of knockout clones. **h** Detection of indels in p65 and EZH2 knockout cells. **i** DNA sequences for each of the PCR products from p65 and EZH2 clones. Cut sequences are shown in brackets and loxP sequences are in red. **j** Western blot detected the effect of p65 and EZH2 KO. **k, l** Statistics of the cell cycle in Fig. 5 and Fig. S5. **m, n** apoptosis assays in WT, EZH2 KO and EZH2 KO with EZH2 ectopic overexpression LN229 and U251 cells transfected with PCDH-p65 or PCDH vector. Values are expressed as the mean±SD from three experiments. Values are expressed as the means±SD from three experiments, and the asterisk indicates the statistical significance compared to the controls (*, $p < 0.05$, **, $p < 0.01$, ***, $p < 0.001$, ****, $p < 0.0001$).

Fig. S6 Related to Fig. 6 glioblastoma can be molecularly stratified for risk using NF-κB and EZH2. **a,b** Kaplan–Meier survival analysis of p65 and EZH2 in CGGA database. **c** Pearson correlation coefficient (r) and p-value (p) between EZH2 and p65 mRNA expression (in $\log_2$). **d-k** Kaplan–Meier survival analysis of age, gender, EGFR expression, WHO grades. **l** The differential expression of p65 between glioblastoma and normal tissues. **m** The differential expression of EZH2 between glioblastoma and normal tissues. Values were expressed as the means±SD from three experiments, and the asterisk indicates the statistical significance compared to the controls (*, $p < 0.05$, **, $p < 0.01$, ***, $p < 0.001$, ****, $p < 0.0001$).

Fig. S7 Related to Fig. 7 Synergistic effect induced by inhibition of EZH2 and NF-κB in glioblastoma. **a** LN229 and U251 cell lines were treated with increasing doses of EPZ-6438 in combination with increasing doses of CAPE for 24 hours, and proliferation was monitored by CCK8-kit. Combenefit calculated cell availability and heatmaps. **b** Semi Log dose-response curves and calculated EC50 values of EPZ-6438 and CAPE. **c~e** NF-κB inhibitor CAPE combined with EPZ-6438. Colony formation, cell cycle and apoptosis assay were performed in LN229 and U251 cell lines. **f** Subcutaneously implanted mice were treated with EPZ-6438, CAPE or combination. Images of representative tumors for every group at day 28. Scale bar, 1 cm. Values are expressed as the means±SD from three experiments, and the asterisk indicates the statistical significance compared to the controls (*, $p < 0.05$, **, $p < 0.01$, ***, $p < 0.001$, ****, $p < 0.0001$).

Fig. S8 Schematic of the proposed mechanism.

Supplementary figures

Fig. S1

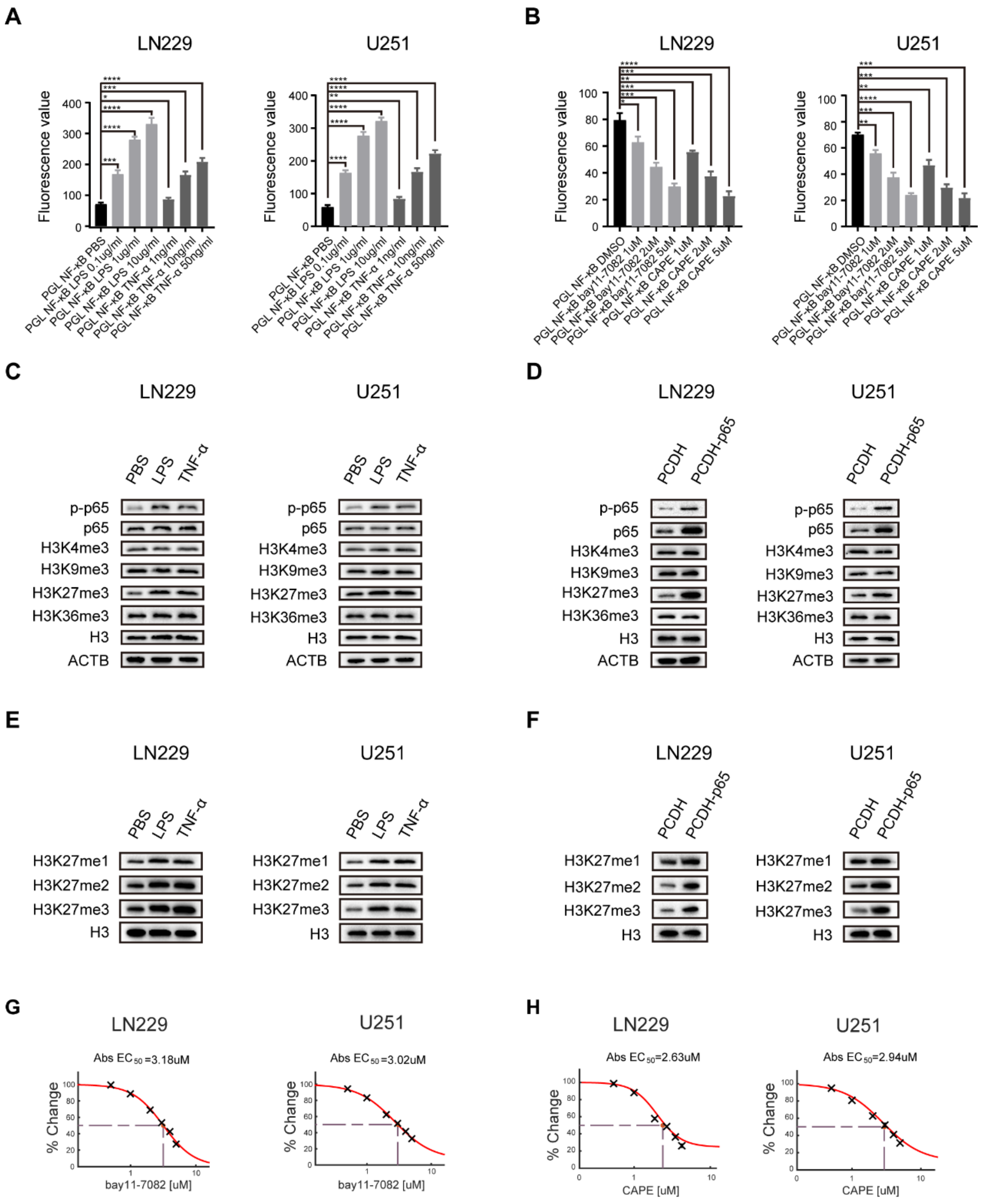

Fig. S2

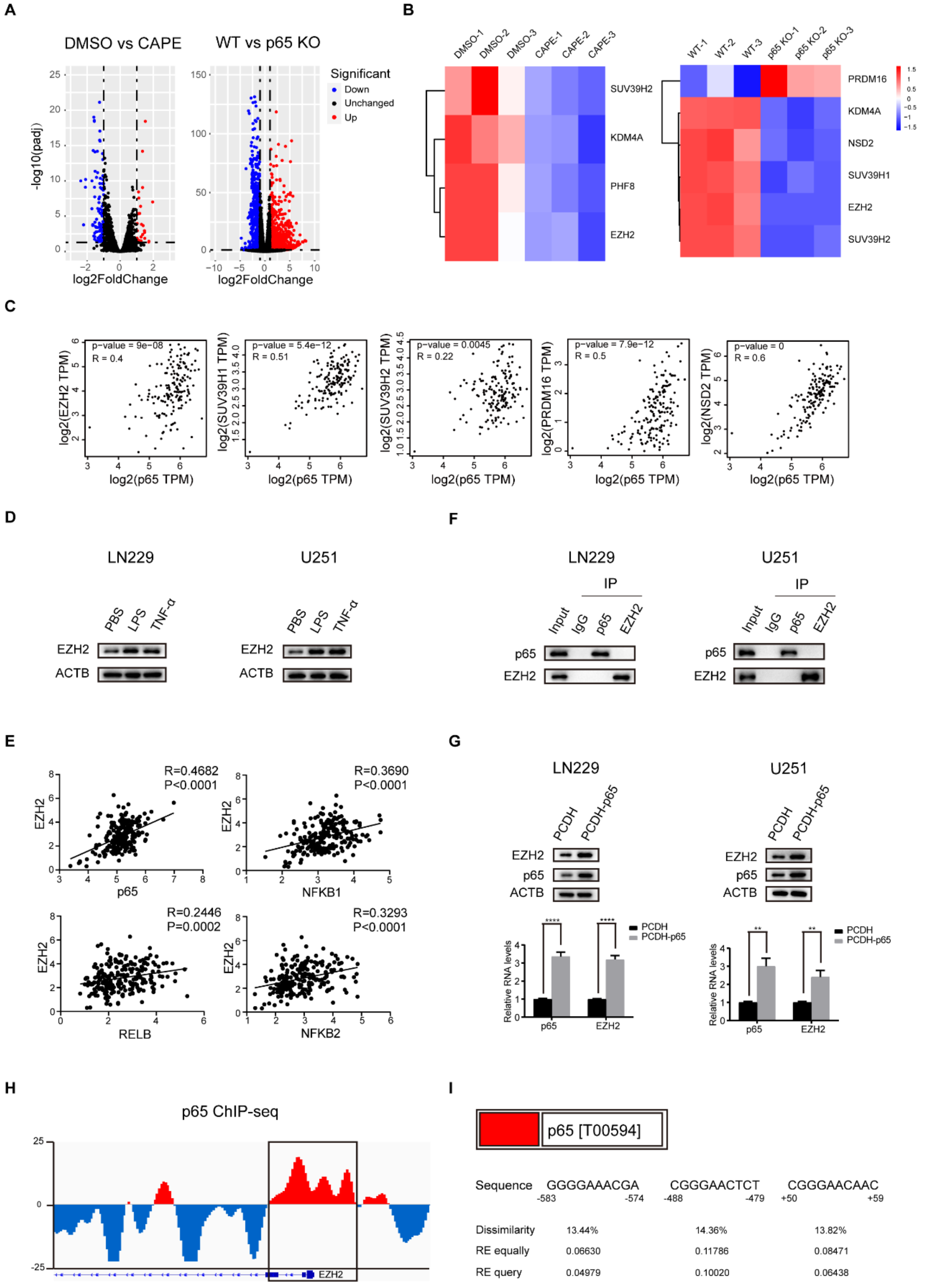

A
DMSO vs CAPE
WT vs p65 KO
-log10(padj)
log2FoldChange
log2FoldChange
Significant
Down
Unchanged
Up
B
DMSO-1
DMSO-2
DMSO-3
CAPE-1
CAPE-2
CAPE-3
SUV39H2
KDM4A
PHF8
EZH2
WT-1
WT-2
WT-3
p65 KO-1
p65 KO-2
p65 KO-3
PRDM16
KDM4A
NSD2
SUV39H1
EZH2
SUV39H2
C
p−value = 9e−08
R = 0.4
log2(EZH2 TPM)
p−value = 5.4e−12
R = 0.51
log2(SUV39H1 TPM)
p−value = 0.0045
R = 0.22
log2(SUV39H2 TPM)
p−value = 7.9e−12
R = 0.5
log2(PRDM16 TPM)
p−value = 0
R = 0.6
log2(NSD2 TPM)
log2(p65 TPM)
D
LN229
U251
PBS
LPS
TNF-α
EZH2
ACTB
F
LN229
U251
IP
Input
IgG
p65
EZH2
p65
EZH2
E
R=0.4682
P<0.0001
R=0.3690
P<0.0001
EZH2
p65
NFKB1
R=0.2446
P=0.0002
R=0.3293
P<0.0001
RELB
NFKB2
G
LN229
U251
PCDH
PCDH-p65
EZH2
p65
ACTB
Relative RNA levels
p65
EZH2
H
p65 ChIP-seq
EZH2
I
p65 [T00594]
Sequence GGGGAAACGA -583 -574 CGGGAACTCT -488 -479 CGGGAACAAC +50 +59
Dissimilarity 13.44% 14.36% 13.82%
RE equally 0.06630 0.11786 0.08471
RE query 0.04979 0.10020 0.06438

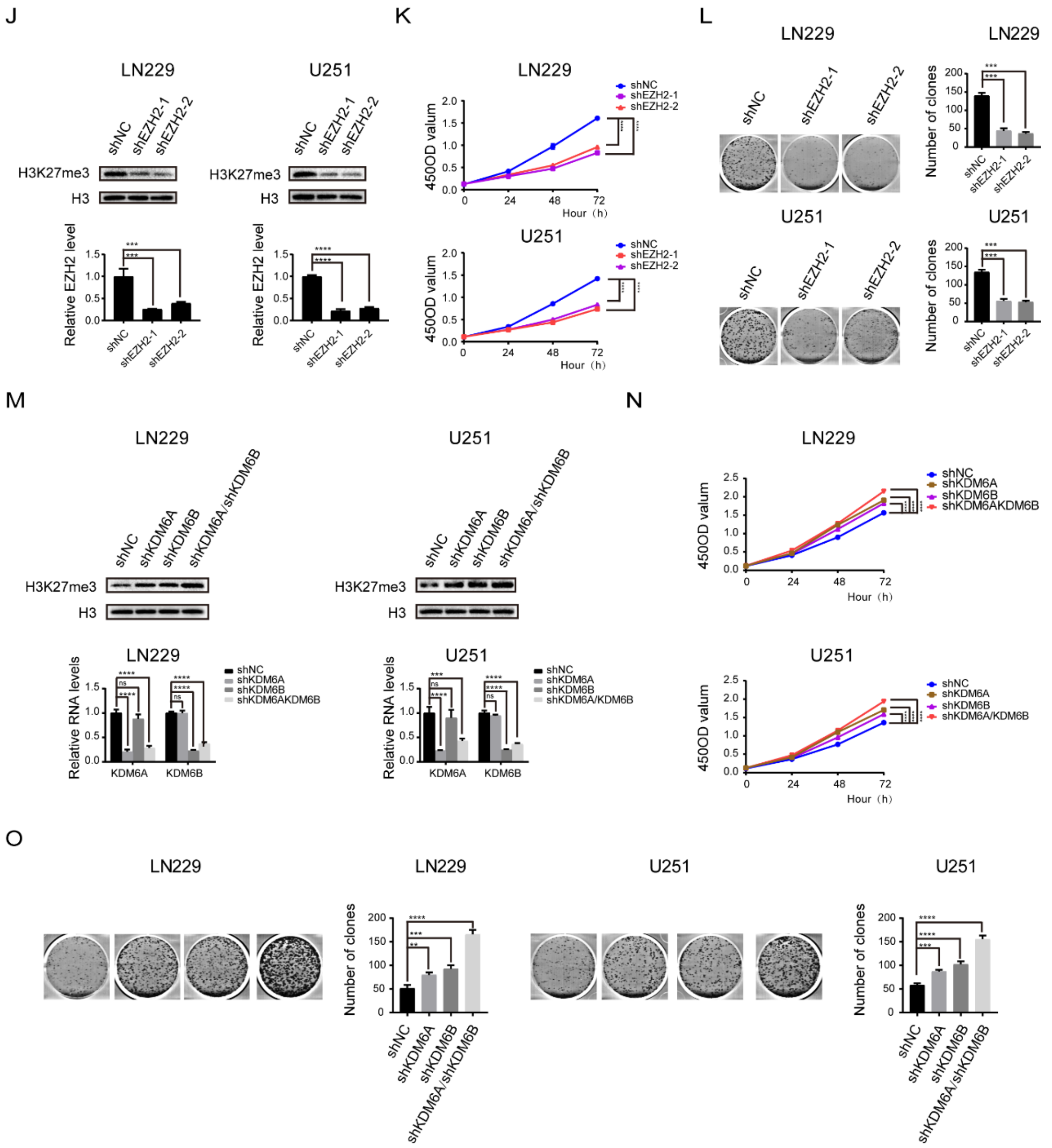

J
K
L
M
N
O
LN229
U251
shNC
shEZH2-1
shEZH2-2
H3K27me3
H3
Relative EZH2 level
450OD valum
Hour（h）
Number of clones
shKDM6A
shKDM6B
shKDM6A/shKDM6B
shKDM6AKDM6B
shKDM6A/KDM6B
Relative RNA levels
KDM6A
KDM6B
ns

Fig. S3

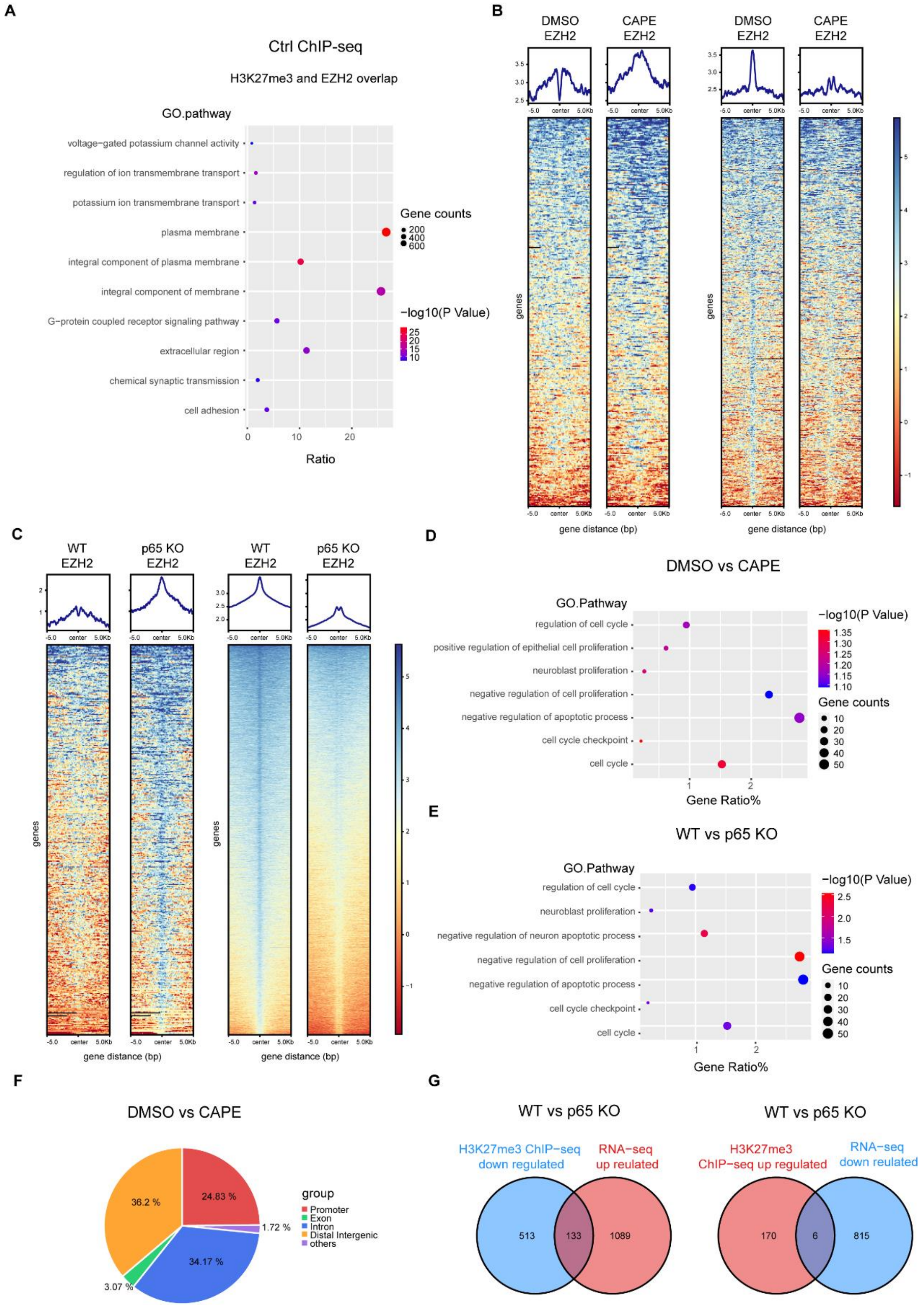
A
Ctrl ChIP-seq
H3K27me3 and EZH2 overlap
GO.pathway
voltage-gated potassium channel activity
regulation of ion transmembrane transport
potassium ion transmembrane transport
plasma membrane
integral component of plasma membrane
integral component of membrane
G-protein coupled receptor signaling pathway
extracellular region
chemical synaptic transmission
cell adhesion
Ratio
Gene counts
-log10(P Value)
B
DMSO EZH2
CAPE EZH2
genes
gene distance (bp)
C
WT EZH2
p65 KO EZH2
genes
gene distance (bp)
D
DMSO vs CAPE
GO.Pathway
regulation of cell cycle
positive regulation of epithelial cell proliferation
neuroblast proliferation
negative regulation of cell proliferation
negative regulation of apoptotic process
cell cycle checkpoint
cell cycle
Gene Ratio%
-log10(P Value)
Gene counts
E
WT vs p65 KO
GO.Pathway
regulation of cell cycle
neuroblast proliferation
negative regulation of neuron apoptotic process
negative regulation of cell proliferation
negative regulation of apoptotic process
cell cycle checkpoint
cell cycle
Gene Ratio%
-log10(P Value)
Gene counts
F
DMSO vs CAPE
36.2 %
24.83 %
1.72 %
34.17 %
3.07 %
group
Promoter
Exon
Intron
Distal Intergenic
others
G
WT vs p65 KO
H3K27me3 ChIP-seq down regulated
RNA-seq up reulated
513
133
1089
WT vs p65 KO
H3K27me3 ChIP-seq up regulated
RNA-seq down reulated
170
6
815

Fig. S4

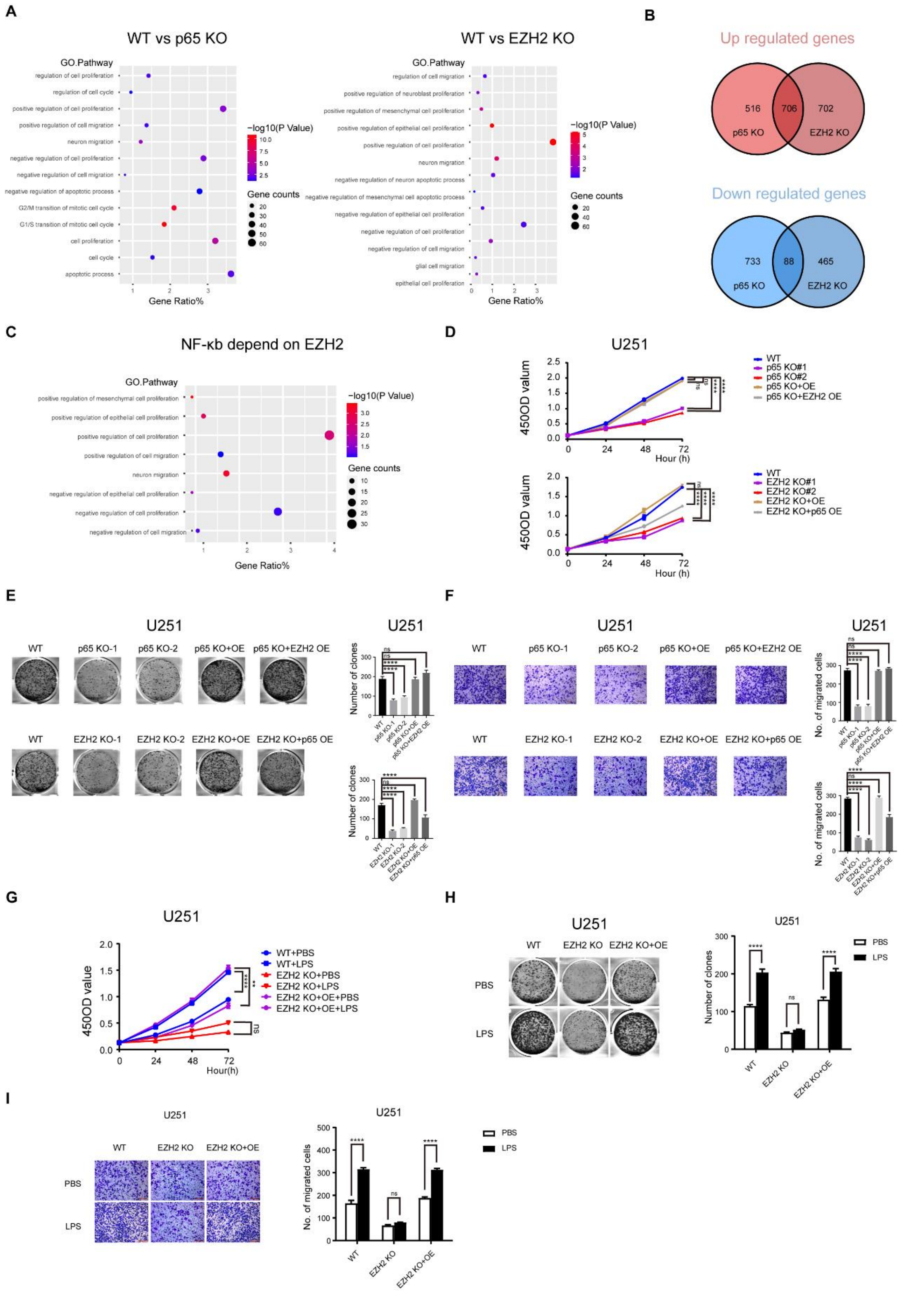
A
WT vs p65 KO
WT vs EZH2 KO
GO.Pathway
Gene Ratio%
-log10(P Value)
Gene counts
B
Up regulated genes
516
706
702
p65 KO
EZH2 KO
Down regulated genes
733
88
465
p65 KO
EZH2 KO
C
NF-κb depend on EZH2
D
U251
450OD valum
Hour (h)
WT
p65 KO#1
p65 KO#2
p65 KO+OE
p65 KO+EZH2 OE
EZH2 KO#1
EZH2 KO#2
EZH2 KO+OE
EZH2 KO+p65 OE
E
U251
Number of clones
F
No. of migrated cells
G
450OD value
Hour(h)
WT+PBS
WT+LPS
EZH2 KO+PBS
EZH2 KO+LPS
EZH2 KO+OE+PBS
EZH2 KO+OE+LPS
H
PBS
LPS
I

Fig. S5

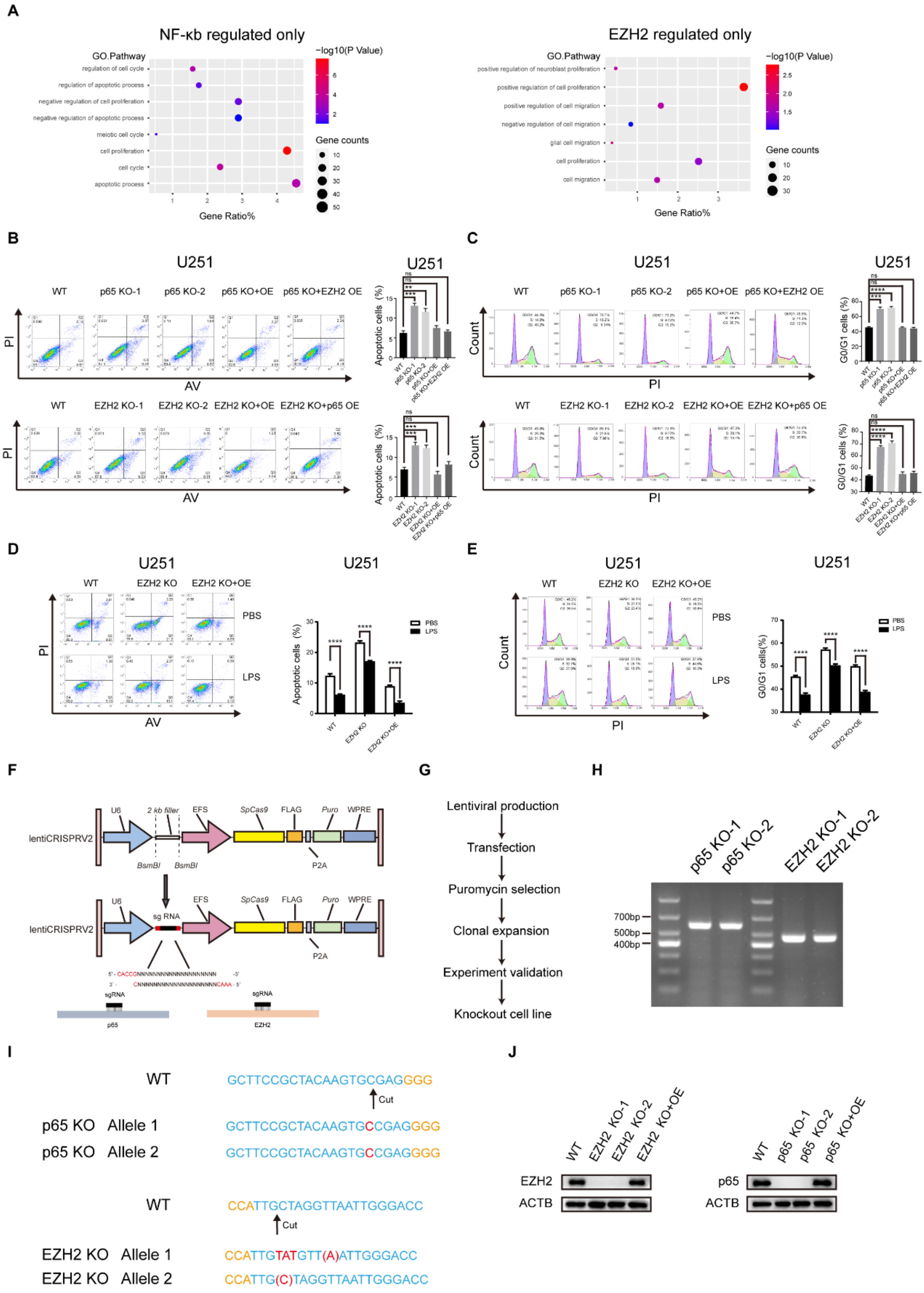
A
NF-κb regulated only
EZH2 regulated only
GO.Pathway
−log10(P Value)
Gene counts
Gene Ratio%
B
U251
AV
PI
Apoptotic cells (%)
C
Count
G0/G1 cells (%)
D
PBS
LPS
E
F
lentiCRISPRV2
G
Lentiviral production
Transfection
Puromycin selection
Clonal expansion
Experiment validation
Knockout cell line
H
p65 KO-1
p65 KO-2
EZH2 KO-1
EZH2 KO-2
700bp
500bp
400bp
I
WT
GCTTCCGCTACAAGTGCGAGGGG
Cut
p65 KO Allele 1
GCTTCCGCTACAAGTGCCCGAGGGG
p65 KO Allele 2
GCTTCCGCTACAAGTGCCCGAGGGG
WT
CCATTGCTAGGTTAATTGGGACC
Cut
EZH2 KO Allele 1
CCATTGTATGTT(A)ATTGGGACC
EZH2 KO Allele 2
CCATTG(C)TAGGTTAATTGGGACC
J
EZH2
ACTB
p65

K

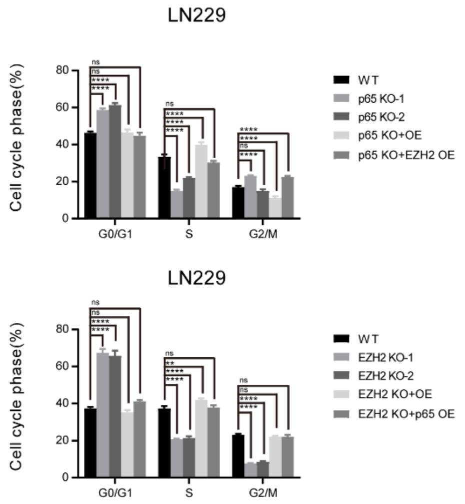
LN229
Cell cycle phase(%)
G0/G1
S
G2/M
WT
p65 KO-1
p65 KO-2
p65 KO+OE
p65 KO+EZH2 OE
LN229
Cell cycle phase(%)
WT
EZH2 KO-1
EZH2 KO-2
EZH2 KO+OE
EZH2 KO+p65 OE

L

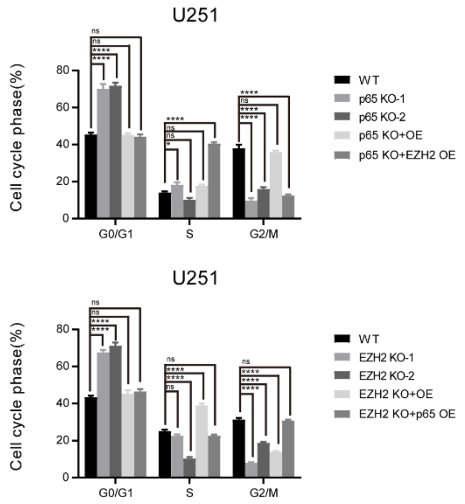
U251
Cell cycle phase(%)
G0/G1
S
G2/M
WT
p65 KO-1
p65 KO-2
p65 KO+OE
p65 KO+EZH2 OE
U251
Cell cycle phase(%)
WT
EZH2 KO-1
EZH2 KO-2
EZH2 KO+OE
EZH2 KO+p65 OE

M

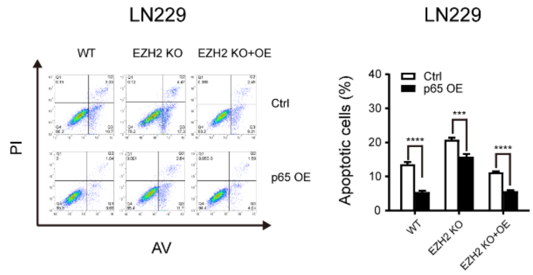
LN229
WT
EZH2 KO
EZH2 KO+OE
Ctrl
p65 OE
PI
AV
LN229
Apoptotic cells (%)
Ctrl
p65 OE
WT
EZH2 KO
EZH2 KO+OE

N

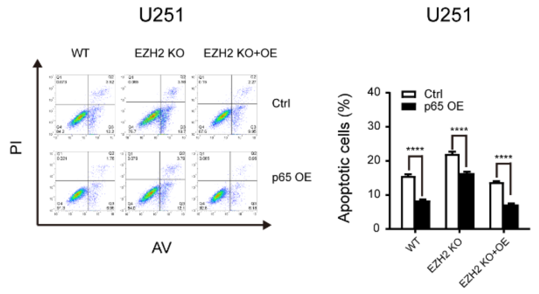
U251
WT
EZH2 KO
EZH2 KO+OE
Ctrl
p65 OE
PI
AV
U251
Apoptotic cells (%)
Ctrl
p65 OE
WT
EZH2 KO
EZH2 KO+OE

Fig. S6

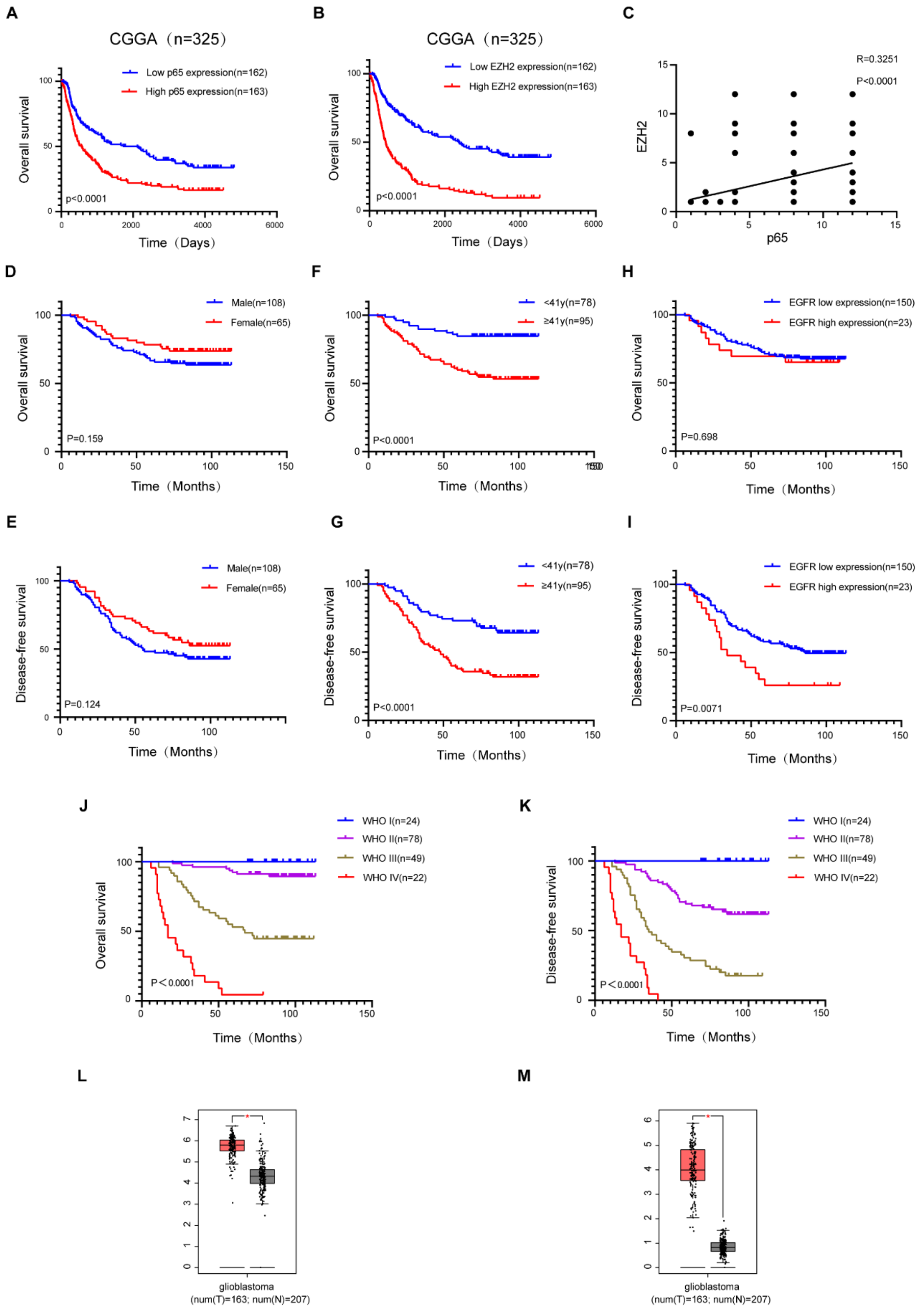
A
CGGA（n=325）
Low p65 expression(n=162)
High p65 expression(n=163)
Overall survival
p<0.0001
Time（Days）
B
CGGA（n=325）
Low EZH2 expression(n=162)
High EZH2 expression(n=163)
Overall survival
p<0.0001
Time（Days）
C
R=0.3251
P<0.0001
EZH2
p65
D
Male(n=108)
Female(n=65)
Overall survival
P=0.159
Time（Months）
F
<41y(n=78)
≥41y(n=95)
Overall survival
P<0.0001
Time（Months）
H
EGFR low expression(n=150)
EGFR high expression(n=23)
Overall survival
P=0.698
Time（Months）
E
Male(n=108)
Female(n=65)
Disease-free survival
P=0.124
Time（Months）
G
<41y(n=78)
≥41y(n=95)
Disease-free survival
P<0.0001
Time（Months）
I
EGFR low expression(n=150)
EGFR high expression(n=23)
Disease-free survival
P=0.0071
Time（Months）
J
WHO I(n=24)
WHO II(n=78)
WHO III(n=49)
WHO IV(n=22)
Overall survival
P＜0.0001
Time（Months）
K
WHO I(n=24)
WHO II(n=78)
WHO III(n=49)
WHO IV(n=22)
Disease-free survival
P＜0.0001
Time（Months）
L
glioblastoma
(num(T)=163; num(N)=207)
M
glioblastoma
(num(T)=163; num(N)=207)

Fig. S7

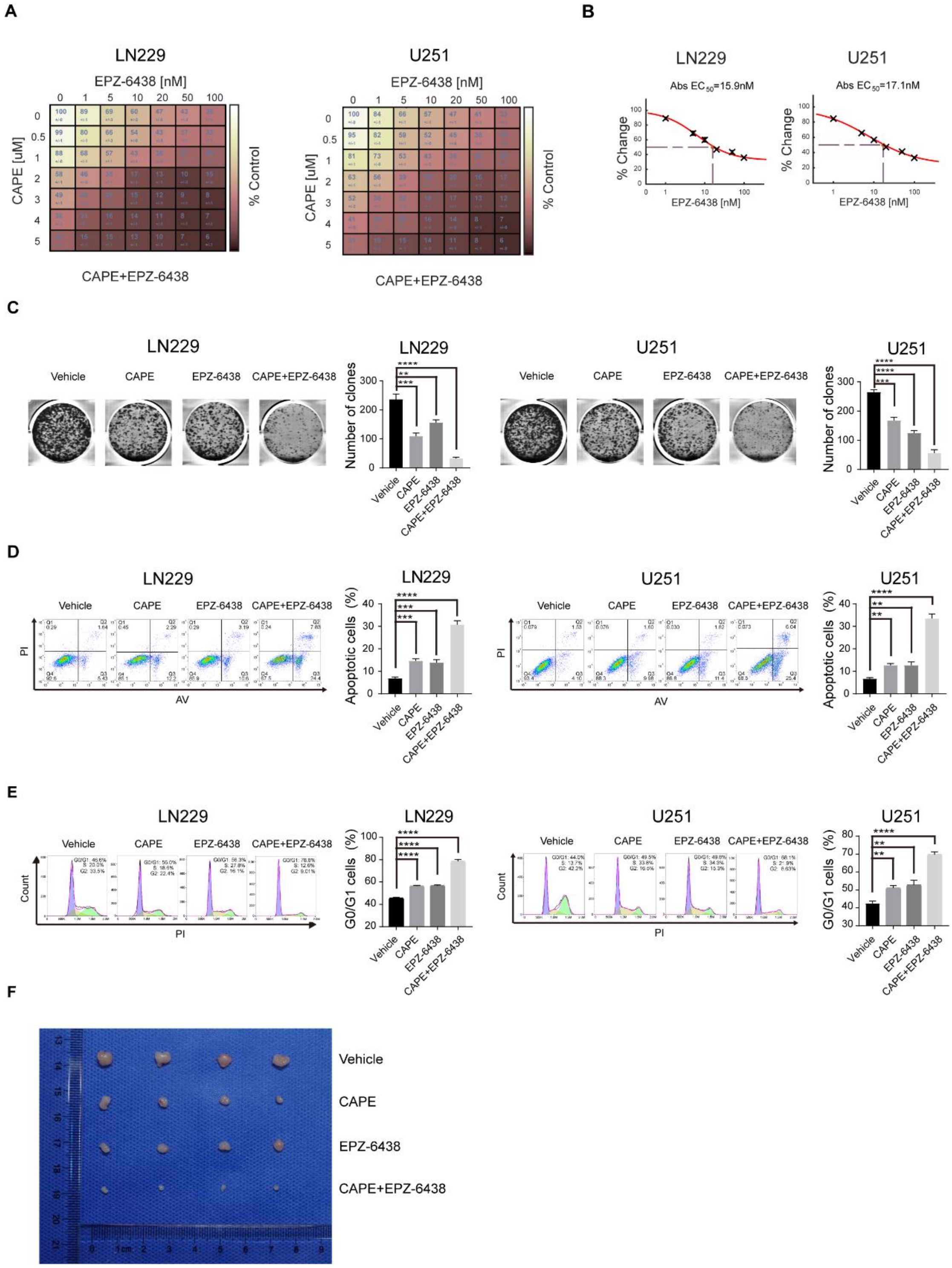
A
LN229
EPZ-6438 [nM]
CAPE [uM]
% Control
CAPE+EPZ-6438
U251
EPZ-6438 [nM]
CAPE [uM]
% Control
CAPE+EPZ-6438
B
LN229
Abs EC_{50}=15.9nM
% Change
EPZ-6438 [nM]
U251
Abs EC_{50}=17.1nM
% Change
EPZ-6438 [nM]
C
LN229
Vehicle
CAPE
EPZ-6438
CAPE+EPZ-6438
Number of clones
U251
D
LN229
PI
AV
Apoptotic cells (%)
U251
E
LN229
Count
PI
G0/G1 cells (%)
U251
F
Vehicle
CAPE
EPZ-6438
CAPE+EPZ-6438

Fig. S8

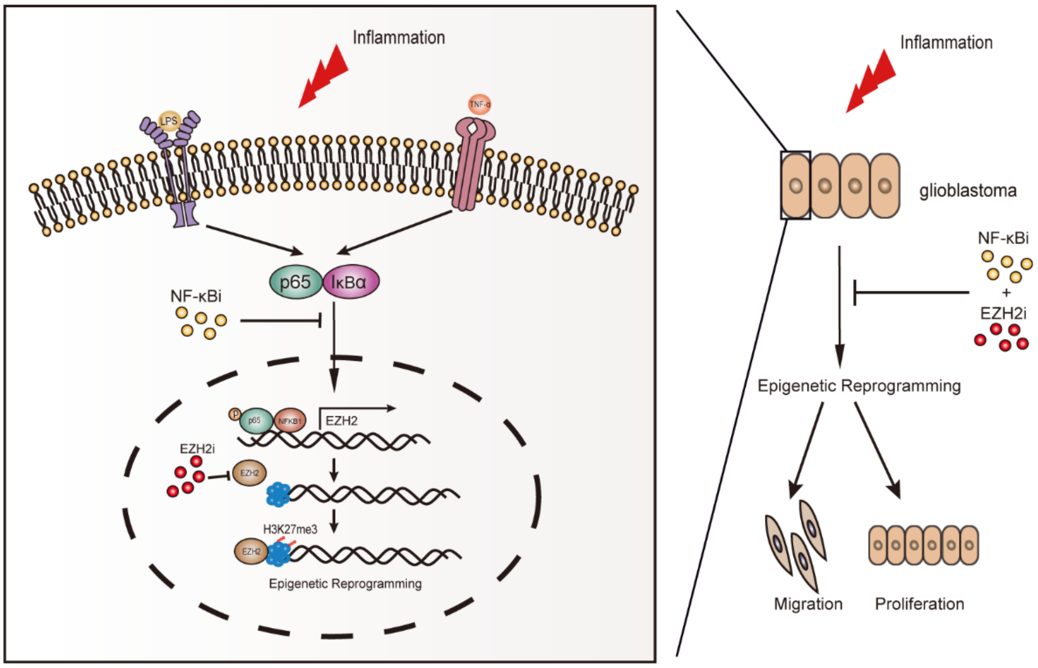

Inflammation
LPS
TNF-α
p65
IκBα
NF-κBi
P
p65
NFKB1
EZH2
EZH2i
EZH2
H3K27me3
EZH2
Epigenetic Reprogramming
Inflammation
glioblastoma
NF-κBi
+
EZH2i
Epigenetic Reprogramming
Migration
Proliferation

Supplementary tables

Table S1: Clinicopathologic characteristics in 173 glioma patients

**Characteristics**	**Number**
Sex	
male	108 (62.4%)
female	65 (37.6%)
Age	
<41y	78 (45.1%)
≥41y	95 (54.9%)
WHO grades	
I	24 (13.9%)
II	78 (45.1%)
III	49 (28.3%)
IV	22 (12.7%)
EGFR expression	
High	23 (13.3%)
low	150(86.7%)
p65 expression	
High	94 (54.3%)
low	79 (45.7%)
EZH2 expression	
High	40(23.1%)
low	133 (76.9%)

Table S2: Relations between the level of p65 or EZH2 expression and clinicopathologic characteristics in 173 glioma patients

**Characteristics**	**p65/EZH2 expression**			**P-value**
	**High/High (%)**	**High/Low (%)**	**Low/Low (%)**	
Overall	31(17.9%)	72(41.6%)	70(40.5%)	
Sex				0.495
male	22(71.0%)	45(62.5%)	41(58.6%)	
female	9(29.0%)	27(37.5%)	29(41.4%)	
Age				**<0.0001**
<41y	6(19.4%)	26(36.1%)	46(65.7%)	
≥41y	25(80.6%)	46(63.9%)	24(34.3%)	
WHO grades				**<0.0001**
I	1(3.2%)	3(4.2%)	20(28.6%)	
II	10(32.3%)	37(51.4%)	31(44.3%)	
III	11(35.5%)	23(31.9%)	15(21.4%)	
IV	9(29%)	9(12.5%)	4(5.7%)	
EGFR expression				**<0.0001**
High	10(32.3%)	11(15.3%)	2(2.9%)	
low	21(67.7%)	61(84.7%)	68(97.1%)	

Table S3: Univariate analysis of five-year overall survival and five-year disease-free survival on different clinicopathological factors by Kaplan–Meier method.

**Variable**	**5-OS (%)**	**Log-rank test (P-value)**	**5-DFS (%)**	**Log-rank test (P-value)**
Sex		0.159		0.124
male	66.7%		48.1%	
female	78.4%		63.1%	
Age		**<0.0001**		**<0.0001**
<41y	84.6%		73.1%	
≥41y	60.0%		37.9%	
WHO grades		**<0.0001**		**<0.0001**
I	100%		100%	
II	92.3%		69.2%	
III	53.1%		30.6%	
IV	4.5%		0%	
EGFR expression		0.698		**0.007**
High	69.6%		26.1%	
low	71.3%		58.0%	
Risk group		**0.029**		**<0.0001**
High-risk group	58.1%		25.8%	
Intermediate-risk group	68.1%		48.6%	
Low-risk group	80.0%		71.4%	

Note:

Abbreviations: 5-OS, five-year overall survival; 5-DFS, five-year disease-free survival;

Table S4: RT-PCR and qPCR primers

**Primers**	**sequence（5'→3'）**
ACTB-F	ATGTGGCCGAGGACTTTGATT
ACTB-R	AGTGGGGTGGCTTTTAGGATG
P65-F	CTATAGAAGAGCAGCGTGGGG
P65-R	TCACTCGGCAGATCTTGAGC
EZH2-F	GCAGCCTTGTGACAGTTCGT
EZH2-R	GCGGCTCCACAAGTAAGACA
Phf8-F	AAGGTTCAACGTCCCTGCTC
Phf8-R	GATAGGCTGGGCTCTTTCCC
NSD2-F	CCTATTGCTGCACGTCAGGT
NSD2-R	CTCTGATGGGTTGCGGTCAT
Prdm16-F	ATGCCGACTTTTGGGAAGGG
Prdm16-R	GTGGAGAGGAGTGTCTTCGG
Kdm4a-F	GGCTTTGGGCTGTAGATTCCT
Kdm4a-R	CGATGAGCTCCTTGGGATTCA
SUV39H1-F	TGATGAGGGGCGGATTGAAC
SUV39H1-R	CCCCACCATTGGTCAAGTCA
SUV39H2-F	GTGCCTTGCCTAGTTTCACT
SUV39H2-R	AGAATCTGGCCATCCTTTCCA
CHIP-BS1-F	CTCCCCGGGCACCACTA
CHIP-BS1-R	AGTTCAAAACTCGGGGGTGG
CHIP-BS2-F	TAAAACCGTTACCACCCCCG
CHIP-BS2-R	CCTCTCAGGAAGGCGGTGT
CHIP-BS3-F	GGGGCCAAATAAAAGCGATG
CHIP-BS3-R	GCGTTACCTTCGTCCCG

Table S5: plasmid primers

**Primers**	**sequence（5'→3'）**
NheI-p65-PCDH-F	CTAGCTAGCATGGACGAACTGTTCCCCCT
BamHI-p65-PCDH-R	CGCGGATCCTTAGGAGCTGATCTGACTCAG
NheI-EZH2-PCDH-F	CTAGCTAGCATGGGCCAGACTGGGAAGAA
BamHI-EZH2-PCDH-R	CGCGGATCCTCAAGGGATTTCCATTTCTCTT
EZH2-promoter-F1	CTCCCCGGGCACCACTA
EZH2-promoter-F2	TACCACCCCCGAGTTTTGAA
EZH2-promoter-F3	CCGGTGGGACTCAGAAGGCA
EZH2-promoter-F4	TTTGTAGGCGTGCGGGGG
EZH2-promoter-R	CCGAAGCTCACAGCTCCTTC
EZH2-BS2-F1	ctggcctaactggccggtaccCTCCCCGGGCACCACTAG
EZH2-BS2-R1	ttggcgggaaccggcgccgcGGCGGGGGAGGGGCGG
EZH2-BS2-F2	GCGGCGCCGGTTCCCG
EZH2-BS2-R2	ccagatcttgatatcctcgagCCGAAGCTCACAGCTCCTTC
p65-sg1 F	CACCGCTTCCGCTACAAGTGCGAG
p65-sg1 R	AAACCTCGCACTTGTAGCGGAAGC
p65-sg2 F	CACCGAAGGCACAGCAATGCGTCG
p65-sg2 R	AAACCGACGCATTGCTGTGCCTTC
EZH2-sg1 F	CACCGCCGGAGCTCAGGGGGATTT
EZH2-sg1 R	AAACAAATCCCCCTGAGCTCCGGC
EZH2-sg2 F	CACCGGTCCCAATTAACCTAGCAA
EZH2-sg2 R	AAACTTGCTAGGTTAATTGGGACC

Abbreviations

NF-κB: Nuclear factor kappa B; EZH2: Enhancer of zeste 2; SPF: Specific pathogen free; CAPE: Caffeic acid phenethyl ester; ATCC: American Type Culture Collection; STR: Short tandem repeat; GO: gene ontology.
